# Developmental Regulation of the *Tetrahymena thermophila* Origin Recognition Complex

**DOI:** 10.1371/journal.pgen.1004875

**Published:** 2015-01-08

**Authors:** Po-Hsuen Lee, Xiangzhou Meng, Geoffrey M. Kapler

**Affiliations:** 1 Department of Molecular and Cellular Medicine, Texas A&M University Health Science Center, College Station, Texas, United States of America; 2 Department of Biochemistry and Biophysics, Texas A&M University, College Station, Texas, United States of America; University of Minnesota, United States of America

## Abstract

The *Tetrahymena thermophila* DNA replication machinery faces unique demands due to the compartmentalization of two functionally distinct nuclei within a single cytoplasm, and complex developmental program. Here we present evidence for programmed changes in ORC and MCM abundance that are not consistent with conventional models for DNA replication. As a starting point, we show that ORC dosage is critical during the vegetative cell cycle and development. A moderate reduction in Orc1p induces genome instability in the diploid micronucleus, aberrant division of the polyploid macronucleus, and failure to generate a robust intra-S phase checkpoint response. In contrast to yeast ORC2 mutants, replication initiation is unaffected; instead, replication forks elongation is perturbed, as Mcm6p levels decline in parallel with Orc1p. Experimentally induced down-regulation of ORC and MCMs also impairs endoreplication and gene amplification, consistent with essential roles during development. Unexpectedly Orc1p and Mcm6p levels fluctuate dramatically in developing wild type conjugants, increasing for early cycles of conventional micronuclear DNA replication and macronuclear anlagen replication (endoreplication phase I, rDNA gene amplification). This increase does not reflect the DNA replication load, as much less DNA is synthesized during this developmental window compared to vegetative S phase. Furthermore, although Orc1p levels transiently increase prior to endoreplication phase II, Orc1p and Mcm6p levels decline when the replication load increases and unconventional DNA replication intermediates are produced. We propose that replication initiation is re-programmed to meet different requirements or challenges during the successive stages of *Tetrahymena* development.

## Author Summary

The Origin Recognition Complex is required for site-specific replication initiation in eukaryotic chromosomes. Null mutations are lethal in yeast and metazoa, and hypomorphs induce genome instability, a hallmark of cancer. We exploited the unique biology of *Tetrahymena* to explore ORC's role in conventional and alternative replication programs. Modest experimental down-regulation of ORC1 induces genome instability in vegetative growing *Tetrahymena*, and diminishes the capacity to support developmentally regulated endoreplication and gene amplification, consistent with essential roles in all of these processes. ORC mutants fail to activate the ATR checkpoint response, and are compromised in their ability to elongate existing replication forks. Remarkably, ORC and MCM levels fluctuate in unexpected ways during wild type development. Most notably, programmed changes in ORC abundance do not reflect the impending DNA replication load. Relative to the vegetative cell cycle, ORC and MCM levels increase dramatically and are highest early in development, when the replication load is lowest. Conversely, ORC levels are lowest during genome-wide macronuclear endoreplication, when the replication load increases. Endocycling cells generate unconventional replication intermediates that distinguish them from vegetative ORC1 knockdown mutants. The collective data suggest that the dependence on ORC may be relaxed during late stages of macronuclear development.

## Introduction

DNA replication initiates at specific sites in chromosomes, termed origins of replication. While the genomic architecture of replication initiation sites varies widely across the eukaryotic lineage, a conserved feature is their association with the six-subunit Origin Recognition Complex (ORC) [1], [2]. ORC-dependent licensing is required for replication initiation and provides a mechanism to prevent re-replication of chromosomes during S phase. Pre-replicative complex (pre-RC) assembly is mediated by transient interactions between ORC, Cdc6 and Cdt1, which recruit the MCM2-7 complex- the replicative helicase that unwinds the DNA at replication origins and elongating replication forks. Additional factors (Cdc45, GINS) recruit the DNA polymerase machinery to generate pre-initiation complexes. Phosphorylation and/or degradation of Orc1p prevents new pre-RCs from forming on daughter chromosomes [3].

Research with the budding yeast, *Saccharomyces cerevisiae* (Sc), has revealed conserved and unique insights into replication initiation. Sc replicons are short (100–200 bp) and include a conserved 11 bp motif, the ARS consensus sequence (ACS), that is bound by ORC in an ATP-dependent, sequence-specific manner. With an estimated 20,000 Orc2p molecules per cell [4], and 12,000 ACSs, but only 400 replication origins [5], Sc-ORC appears to be in vast excess. Multiple ORC subunits interact with the DNA with the ancestral Orc1p contacting the ACS [6]. Metazoan ORCs exhibit no sequence specificity, and are in modest excess relative to replication origins. In *Drosophila melanogaster*, approximately 30% of in vivo ORC binding sites function as early replication origins [7]. With the exception of Orc1p, the steady state levels of ORC subunits are not differentially regulated in quiescent versus proliferating mammalian cells [8]. Cdc6 and MCM2-7 serve as reliable biomarkers for mammalian cell proliferation [9]. ORC and MCMs are dramatically up-regulated during embryonic development in *Xenopus laevis* to support the rapid S phases prior to the mid-blastula transition [10]. Origin density increases by a factor of ∼10, as replication initiates in coding and non-coding sequences [11]. The onset of zygotic transcription and remodeling of chromatin redirects replication initiation to intergenic regions when ORC protein levels decline.

The ciliated protozoan, *Tetrahymena thermophila*, has served as a useful model for examining the molecular organization of eukaryotic replicons, and studying the genetic and epigenetic control of DNA replication [12]. A distinguishing feature of ciliates is the cohabitation of two functionally distinct nuclei within the same cytoplasm. This arrangement results in the genesis of different autonomous DNA replication programs. The transcriptionally silent diploid micronucleus serves as the reservoir of genetic material that is transmitted from parent to progeny during conjugation. The polyploid macronucleus is actively transcribed throughout the vegetative cell cycle and development. Since the macronucleus is destroyed in progeny [13], a new macronucleus must be generated by differentiation of a post-zygotic micronucleus [13].

The partitioning of chromosomes into two operationally distinct nuclei places unusual demands on DNA replication, including origin licensing and the coordination of checkpoint responses that maintain genome integrity. The temporal order of DNA replication parallels that of higher order chromatin domains in more typical eukaryotic chromosomes [14], [15], in that euchromatic macronuclear chromosomes are replicated prior to replication of the heterochromatic micronuclear genome. However, macronuclear S phase precedes micronuclear S [16], and cytokinesis is coupled to amitotic macronuclear division [17].

Micro- and macronuclear DNA replication programs are uncoupled in conjugating cells. First, meiosis converts a diploid micronucleus into four haploid pronuclei, three of which are degraded [16]. The sole survivor replicates and divides to produce genetically identical migratory and stationary pronuclei, which are reciprocally exchanged between mating partners. The diploid zygotic micronucleus undergoes two rounds of DNA replication and mitosis. Two micronuclei exit the DNA replication program, and two differentiate into macronuclei. During macronuclear development, the five micronuclear chromosomes are extensively remodeled. One third of the genome (including centromeres and retrotransposons) is eliminated by site-specific DNA fragmentation and de novo telomere addition, or removal of internal DNA sequences by breakage and rejoining, generating ∼180 distinct macronuclear chromosomes. Non-coding RNAs generated in the newly formed micronucleus dictate which internal DNA sequences are eliminated from the developing macronucleus [18]. Epigenetic reprogramming of histones converts heterochromatic micronuclear chromosomes into macronuclear euchromatin. Through endoreplication, the copy number of macronuclear chromosomes increases to ∼45 C, and the 21 kb ribosomal DNA minichromosome is amplified to ∼9000 C. Similar to ovarian follicle cells in Drosophila [19], genome-wide endoreplication precedes selective gene amplification. Once development is completed, micro- and macronuclear chromosomes replicate once per vegetative cell cycle. Although amitotic macronuclear chromosomal segregation and unequal nuclear division can generate genic imbalances [20], the copy number of macronuclear chromosomes is maintained in a narrow range. This occurs through the elimination of ‘excess DNA’ in the form of chromatin extrusion bodies, or partial re-replication of macronuclear chromosomes.

Like yeast and metazoa [21], the Tetrahymena Origin Recognition Complex (ORC) specifies where replication initiates, recruiting the MCM2-7 helicase to specific sites in chromosomes. Tetrahymena ORC is unusual in that it contains an integral RNA subunit, designated 26T RNA, which selectively targets ORC to the amplified rDNA origin through Watson-Crick base pairing [22]. 26T RNA is not complementary to regulatory sequences in non-rDNA origins, and ORC is loaded onto rDNA and non-rDNA origins at different stages of the cell cycle [23]. Hence, ORC recruitment and licensing differs for rDNA and non-rDNA origins. ORC and MCM transcript levels are elevated in conjugating cells in parallel with other replication proteins, such as Cdt1, PCNA and DNA polymerase α/primase [24], suggesting that the demands for these proteins increases during development.

DNA damage and replication stress can irreparably harm Tetrahymena chromosomes [25]. While deleterious events may be resolved without the need to arrest the cell cycle, Tetrahymena elicits a robust DNA damage/replication stress checkpoint response when a threshold is exceeded [26]. In yeast and mammals, the intra-S phase checkpoint is triggered by an apical kinase, MEC1 and ATR, respectively [27], [28]. Checkpoint activation leads to the phosphorylation of MCM2-7 helicase subunits, blocking both replication initiation and fork elongation [29]. Tetrahymena encodes a single ATR gene, that has been shown to induce cell cycle arrest and prevent micro- and macronuclear genome instability [26]. ATR is also required for the reorganization of chromosomes during meiosis [30].

In the work presented here, we examine how programmed and experimentally induced changes in the abundance of ORC affect DNA replication and the intra-S phase checkpoint response. We show that the sustained down regulation Orc1p in a macronuclear knockdown mutant induces genome instability in the micro- and macronucleus. Unexpectedly, ORC1 depletion induces defects in replication fork elongation rather than initiation, and fails to activate the intra S-phase checkpoint response. We also document coordinately regulated changes in ORC and MCM protein levels during development, in which the abundance of pre-RC proteins does not correlate with the impending DNA replication load. Finally, we provide evidence for altered replication initiation and/or elongation in endoreplicating macronuclear chromosomes. The collective data demonstrate that the rules for DNA replication change substantially when Tetrahymena exits the vegetative cell cycle and commits to its complex developmental program.

## Results

### Down-regulation of DNA replication components in ORC1 mutants

**Figure 1:**
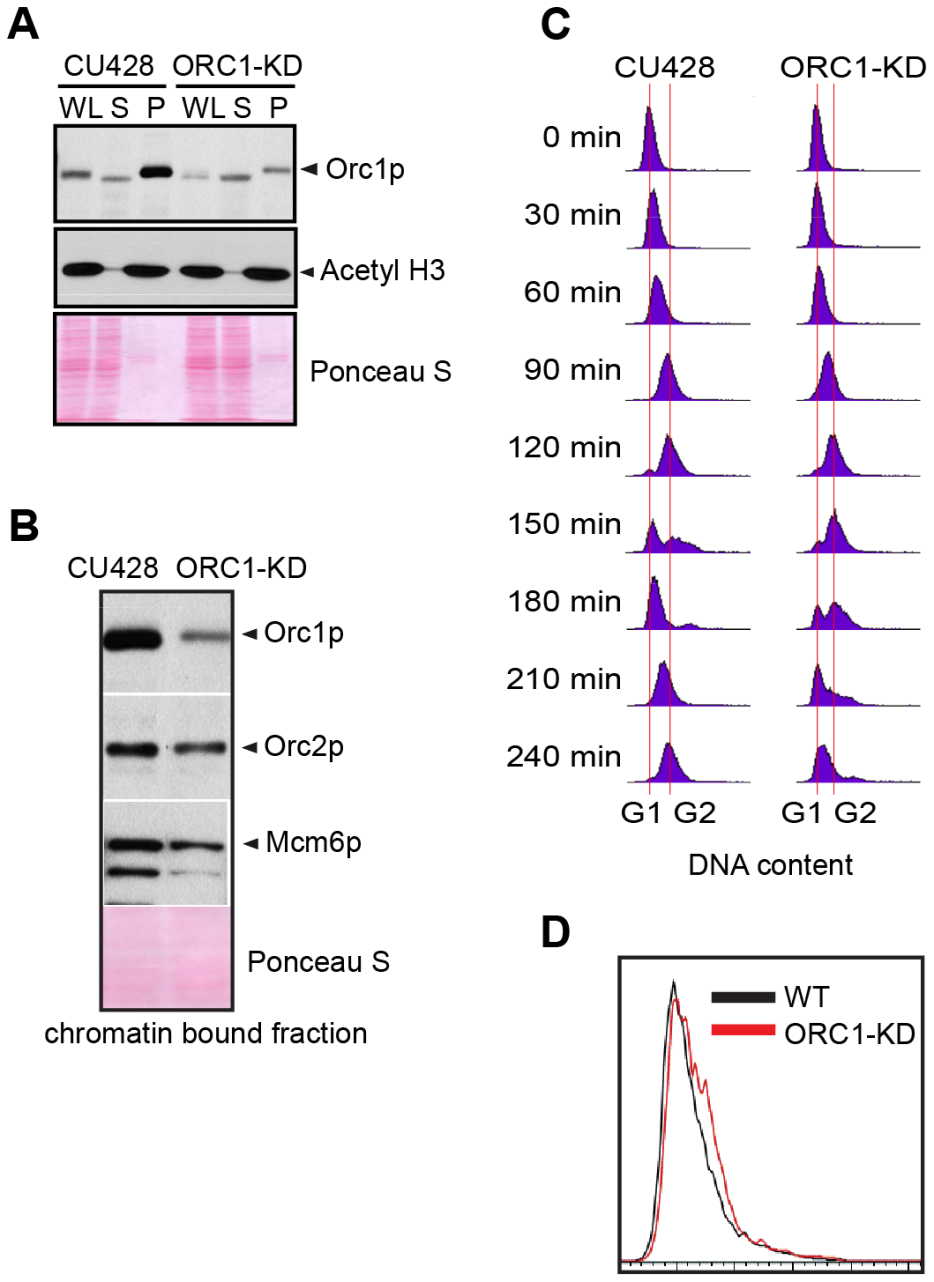
ORC1 depletion induces slow cell cycle progression. (A) Western blot analysis of whole cell lysates (WL), NP-40-extractable soluble fractions (S), and nuclear chromatin-bound pellet fractions (P). Samples were prepared from log phase wild type (CU428) and ORC1 knockdown (ORC1-KD) cells, and immunoblotted with rabbit polyclonal anti-Orc1p, anti-Orc2p, anti-Mcm6p, and anti-acetyl Histone H3 antibodies. Each lane corresponds to proteins derived from 10 µl of cultured cells at density of 2 × 10^5^ cells/ml. Membranes were stained with Ponceau S to visualize total protein loaded in each lane prior to antibody probing. (B) Western blot analysis of chromatin bound pre-RC components in wild type and ORC1 knockdown strains. A Lowry assay was performed to assure that equivalent amounts of protein (20 µg) were loaded in each lane. Due to the different sizes of target proteins, a single membrane was cut into pieces to probe for each target protein. (C) Cell cycle progression of CU428 and ORC1-KD cells as measured by flow cytometry. 0 min corresponds to G1 phase cells isolated by elutriated centrifugation. (D) Flow cytometry analysis of asynchronous, log phase wild type (CU428) and ORC1 knockdown (ORC1-KD) cells. Vegetative growing cell cultures were harvested at late log phase (cell density: 2.5×10^5^ cells/ml).

To evaluate the requirements for ORC during vegetative replication of micro- and macronuclear chromosomes, ORC1 knockdown mutants were generated by targeted gene disruption in the macronucleus (S1 Table). The random segregation of amitotic chromosomes was exploited to obtain phenotypic assortments with reduced dosage of the wild type locus. Using conditions that select for retention of the paromomycin-resistant disruption allele, only a 5-fold reduction of the wild type ORC1 gene (TTHERM_00865050) was achieved in the 45 C macronucleus. A corresponding reduction in Orc1p was observed in the mutant population (Fig. 1A, WL). Even less Orc1p was associated with chromatin in the mutant (Fig. 1A), suggesting that ORC occupancy on chromosomes was ∼10% that of wild type Tetrahymena. Unexpectedly, Orc2p and Mcm6p levels were also reduced in the ORC1 knockdown strain (Fig. 1B). ORC1 knockdown cells grew more slowly than wild type, exhibiting a prolonged S phase (Fig. 1C, compare data for 120–210 min time points), consistent with a defect in DNA replication. Asynchronous cultures contained more cells with a high DNA content (Fig. 1D), and ∼90% of dividing mutant cells underwent asymmetric macronuclear division with lagging chromosomes (S1 Fig., wild type frequency: ∼2%). The collective data are consistent with the failure to fully replicate macronuclear chromosomes.

### ORC1 mutant have a diminished intra-S phase checkpoint response

**Figure 2:**
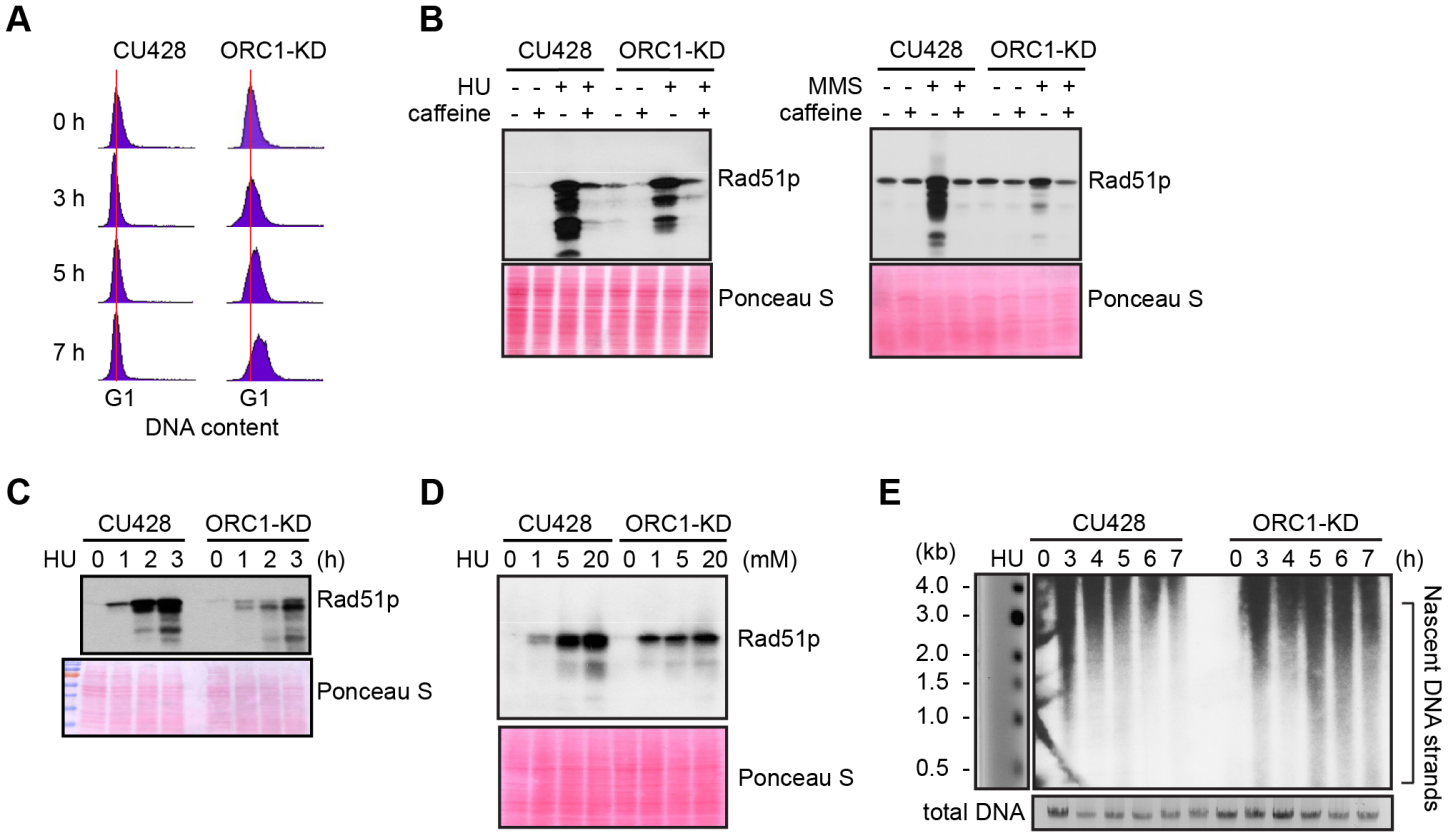
Abrogated intra-S phase checkpoint response in ORC1 knockdown cells. (A) Elutriated G1 phase wild type (CU428) and ORC1 knockdown (ORC1-KD) cells were treated with 20 mM HU and samples were collected at the indicated intervals for flow cytometry analysis. (B) G1 synchronized cells were released into fresh medium containing 20 mM HU or 0.06% MMS +/− 1 mM caffeine (1 mM) for 4 h. Whole cell lysates were subjected to western blot analysis of Rad51p. (C) G1 synchronized cells were incubated in medium containing 20 mM HU. Whole cell lysates were prepared at timed intervals and subjected to western blot analysis with anti-Rad51 antibody. (D) G1 synchronized cells were incubated in the presence of HU (1–20 mM) for 4 h and subjected to western blot analysis. (E) Alkaline gel electrophoresis of nascent DNA strands accumulated under HU treatment. G1 synchronized cells were cultured in 20 mM HU and genomic DNA was isolated at indicated time points. RIs were released under alkaline condition and resolved in a 1% alkaline agarose gel. RIs from the rDNA 5′ NTS origin region were visualized by Southern blot analysis.

S phase induced DNA damage and replication stress trigger an ATR/MEC1 checkpoint response in eukaryotes that prevents new origins from firing and inhibit the elongation of existing replication forks. Both processes are blocked by the reversible phosphorylation of the MCM2-7 complex [29]. To assess DNA replication and DNA damage checkpoint responses, G1 synchronized cultures were treated with hydroxyurea (HU) or methylmethanesulphonate (MMS), respectively. Activation of the ATR checkpoint was monitored by flow cytometry and accumulation of Rad51p, which is rapidly up-regulated [26], [31]. Whereas 20 mM HU inhibited DNA replication in wild type Tetrahymena, the ORC1 knockdown strain entered S phase and continued to synthesize DNA (Fig. 2A), albeit at a slower rate than untreated controls (Fig. 1C). Similar results were obtained for mutant cells treated with 0.06% MMS (S2 Fig.). The inability to inhibit DNA replication is consistent with compromised DNA replication (HU) and DNA damage (MMS) checkpoint responses. ORC1 mutants were less responsive than wild type cells to HU exposure time (Fig. 2C) or dosage (Fig. 2D). While MMS was a more potent inducer of Rad51p production, HU and MMS responses were inhibited by the addition of caffeine (Fig. 2B), consistent with the involvement of ATR.

Alkaline gel electrophoresis was used to visualize nascent DNA strands derived from the rDNA origin region. G1 synchronized cells were incubated for 1–7 h in media containing 20 mM HU and nascent strands were detected by Southern blot analysis with a 5′ non-transcribed spacer (NTS) probe (Fig. 2E). Wild type Tetrahymena generated short nascent strands that gradually chased into high molecular weight species. We interpret this to indicate that fork elongation was slowed down by HU, and that new origin firing was suppressed. In contrast, low molecular weight nascent strands were evident at all time points in HU-treated ORC1 mutant cultures, suggesting that replication initiation was not repressed. The collective data (Fig. 2A–E) indicate that the intra-S phase checkpoint response is compromised in ORC1 knockdown mutant.

### Altered replication fork progression in ORC1 knockdown cells

**Figure 3:**
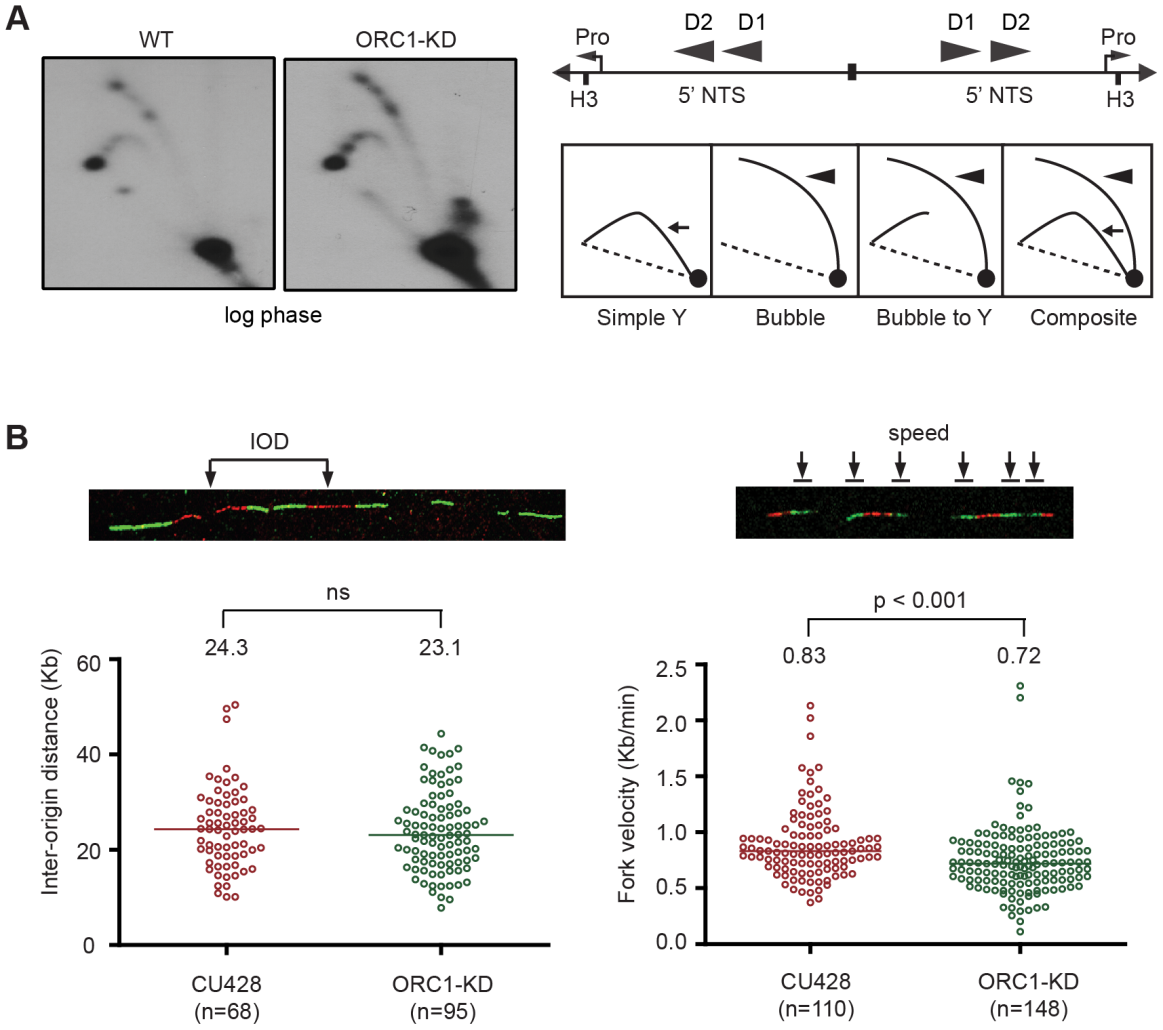
Altered cell cycle distribution and replication fork progression in ORC1 knockdown cells. (A) DNA samples from log phase cultures were subjected to neutral-neutral 2D gel analysis following digestion with *HindIII* and enrichment for RIs on BND cellulose. Left panels: blots were probed with the rDNA 5′ NTS probe (wild type (WT) and ORC1 knockdown (ORC1-KD) strains. Right panel, schematic of the palindromic *HindIII* fragment spanning the two inverted copies of the 5′ NTS, promoter (pro) and replication origins (D1 and D2). (B) Representative images for DNA fibers sequentially labeled with IdU and CldU. Inter-origin distance and fork velocity were measured in log phase CU428 and ORC1-KD cells (see Materials and Methods for details).

The *S. cerevisiae* ORC2-1 mutant is defective in MEC1-dependent checkpoint activation, generating fewer elongating replication forks due to decreased replication initiation. Consequently, the average distance between initiation sites increases from 45 kb to 65 kb [4]. To better understand the impact of ORC1 depletion on the Tetrahymena checkpoint response, two-dimensional gel electrophoresis and DNA combing were use to study DNA replication of the amplified 21 kb rDNA minichromosome and larger non-rDNA macronuclear chromosomes. No differences were evident in the rDNA 5′ NTS replication intermediate (RI) patterns of wild type and mutant strains (Fig. 3A). Bubble-to-Y arc RIs were generated in the mutant and no complete Y arcs were observed, consistent with initiation from known ORC binding sites in the 5′ NTS. The pattern of accumulated RIs on the bubble-to-Y arcs is consistent with the transient pausing of replication forks at conserved PSE elements [32]. Hence, within the limits of resolution, rDNA origin utilization is unaffected. DNA fiber analysis also revealed no change in origin utilization in non-rDNA chromosomes, as the median distance between non-rDNA origins (inter-origin distance, IOD) was unaltered (Fig. 3B; WT IOD: 24.3 kb; ORC1 mutant IOD: 23.1 kb). However, the rate for replication fork elongation (RFE) was significantly reduced in the mutant (Fig. 3B; WT RFE rate: 0.83 kb/min; ORC1 mutant RFE rate: 0.72 kb/min; 14% reduction; p <0.001). These data suggest that the primary defect in the ORC1 mutant occurs downstream of replication initiation.

### Mitotic and meiotic micronuclear genome instability in ORC1 knockdown mutants

**Figure 4:**
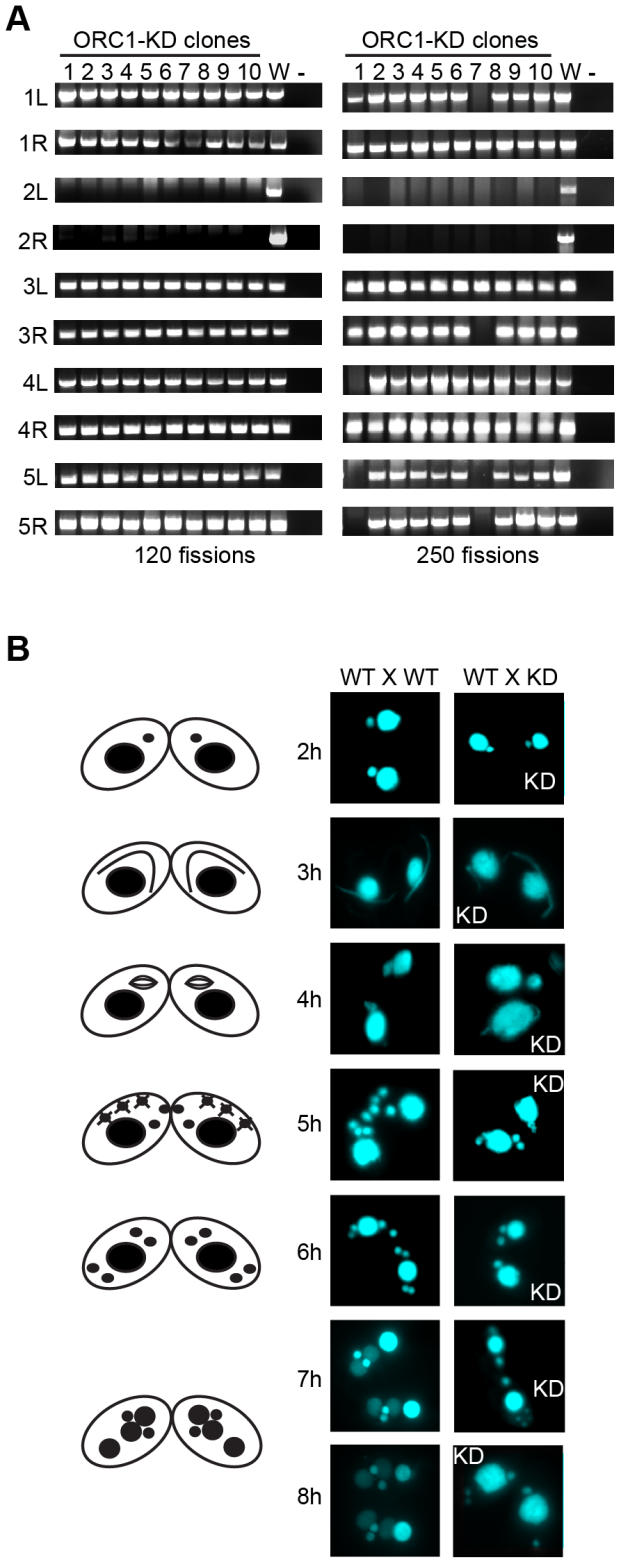
Micronuclear genome instability in ORC1 knockdown cells. (A) ORC1 knockdown (ORC1-KD) cells were propagated following the establishment of clonal lines. Genomic DNA was isolated at 120 and 250 fissions and subjected to PCR amplification with the primer sets that span sites for chromosome breakage sequence (CBS)-mediated chromosome fragmentation in the developing macronucleus. PCR primers derived from the right (R) and left (L) arms of all five micronuclear chromosomes were tested. 1–10, clonal ORC1-KD; W, wild type strain CU427; (−), PCR reactions in the absence of template DNA. (B) Cytological examination of crosses between wild type strains (CU427 X CU428), or CU427 X ORC1-KD. Nuclei were visualized with the DNA staining dye DAPI. Cartoons depict the progression of wild type mating cells during development. 3 h: micronuclear elongation (crescent); 4 h: micronuclear meiosis; 5 h: four haploid meiotic micronuclei, three of which subsequently undergo programmed nuclear death (PND); 6 h: postzygotic division; 7–8 h: macronuclear anlagen differentiation. Prior to mating, one of the parental strains was incubated with mitotracker red dye to determine the identity of each partner in mating pairs.

Since the macronucleus directs all gene expression, the loss of micronuclear chromosomes can be tolerated during the vegetative phase of the life cycle, leading to the genesis of aneuploid micronuclei [33]. To assess micronuclear genome instability in ORC1 knockdown cells, PCR was performed with primer sets that span 10 of the chromosome fragmentation sites used to convert the 5 mitotic micronuclear chromosomes into ∼180 amitotic macronuclear counterparts (one primer set per micronuclear chromosome arm) [26]. Ten clonal ORC1 knockdown lines were generated and propagated for further analysis. All 10 lines failed to produce PCR products at 120 fissions for primer sets diagnostic for the left and right arms of chromosome 2 (Fig. 4A, left panel). Additional micronuclear DNA markers were lost in a subset of clonal lines at 250 fissions (micronuclear chromosome arms 1L, 3R, 4L, 5L, 5R) (Fig. 4A, clones 1 and 7; right panel). While chromosome instability is expected to be stochastic, with new events displaying a clonal inheritance pattern, our ability to detect chromosome loss did not require repeated sub-cloning, as was previously reported for mutations in cis-acting rDNA determinants [34] and trans-acting factors [26].

To assess the effect of Orc1p dosage on meiotic chromosome transmission and subsequent rounds of DNA replication associated with development, early passage knockdown clones that tested positive for all 10 micronuclear chromosome markers were mated to a wild type strain. Strains were pre-incubated with mitotracker dyes to determine the identity of each partner in mating pairs. DAPI was used to follow the fate of micro- and macronuclei. The mutant partner exhibited a temporal delay in formation of the micronuclear crescent, an elongated structure with centromeres and telomeres at opposite ends (Fig. 4B, 3 h and 4 h time points) [31]. Sampling at later time points identified progeny in which post-zygotic micronuclear division was either arrested or developmentally delayed in the mutant. The programmed differentiation of micronuclei into macronuclear anlagen was also perturbed. When post-zygotic defects were detected, both progeny in a mating pair were affected (Fig. 4B, 7 h and 8 h). Since progeny inherit two wild type copies of the ORC1 gene, the macronuclear development program was further examined at the molecular level.

### Endoreplication and gene amplification defects in ORC mutant strains

**Figure 5:**
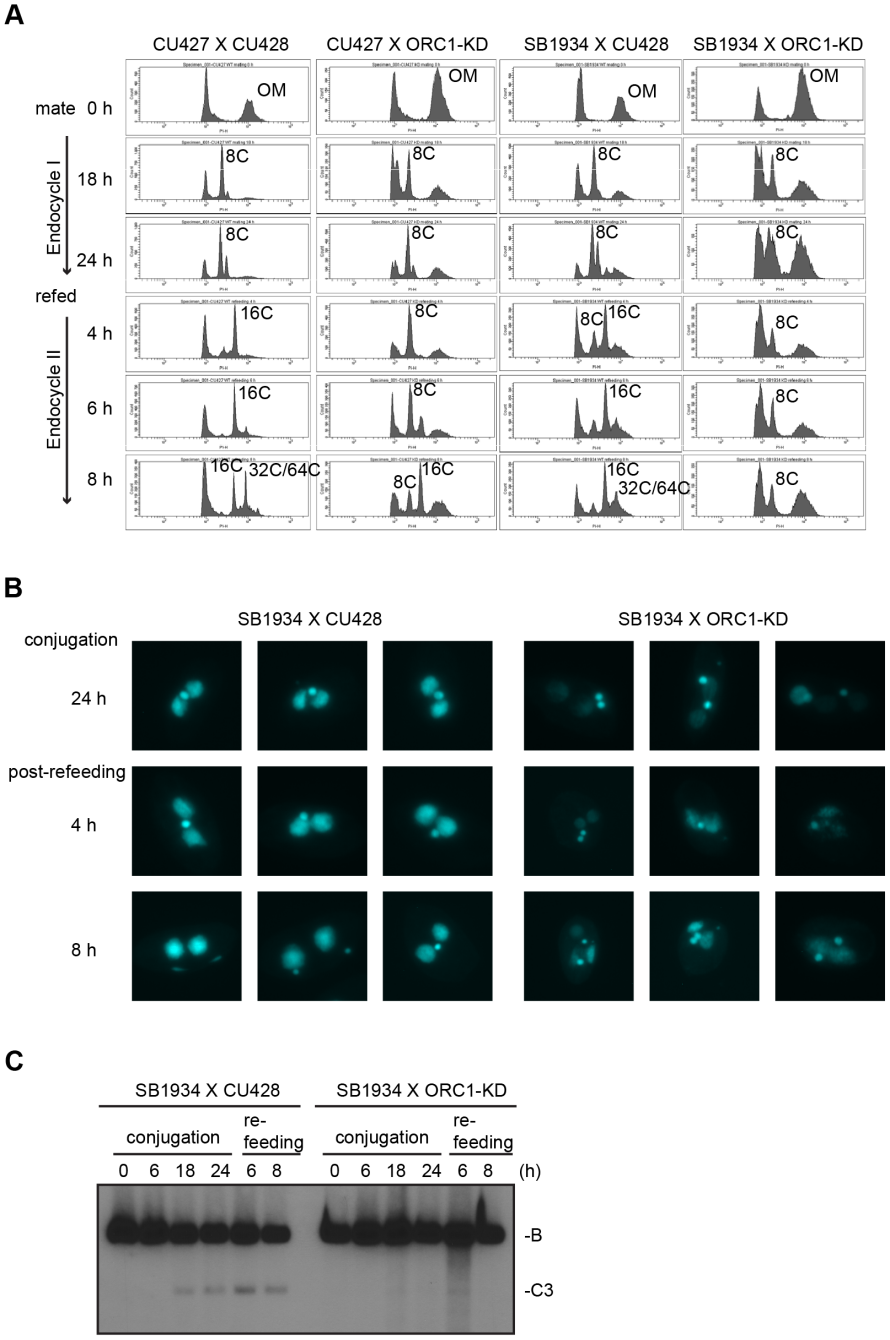
Endoreplication and rDNA amplification during Tetrahymena development. (A) Flow cytometry analysis of matings between wild type and ORC1-KD strains. Nuclei were isolated and stained with propidium iodide for flow cytometric analysis. Wild type strains: CU427, CU428, SB1934. SB1934 is a heterokaryon, with B rDNA in the macronucleus and C3 rDNA in the micronucleus (S1 Table). OM, old parental macronucleus, which is degraded in conjugants. (B) Cytological examination of crosses WT X WT (SB1934 and CU428), and WT X mutant (SB1934 and ORC1-KD) with the DNA staining dye, DAPI. Starved mating cultures were re-fed at 24 h. Three representative images are shown for each time point. (C) Southern blot analysis of C3 rDNA gene amplification during development. Mating between wild type SB1934 or CU428 strains with one another (WT x WT) or with the ORC1-KD strain (WT X mutant) were performed and cells were collected at the indicated time points. DNA was digested with *BamHI* and probed with an rDNA 3′ NTS probe to distinguish macronuclear B (4.0 kb) and C3 (2.5 kb) rDNA alleles.

**Figure 6:**
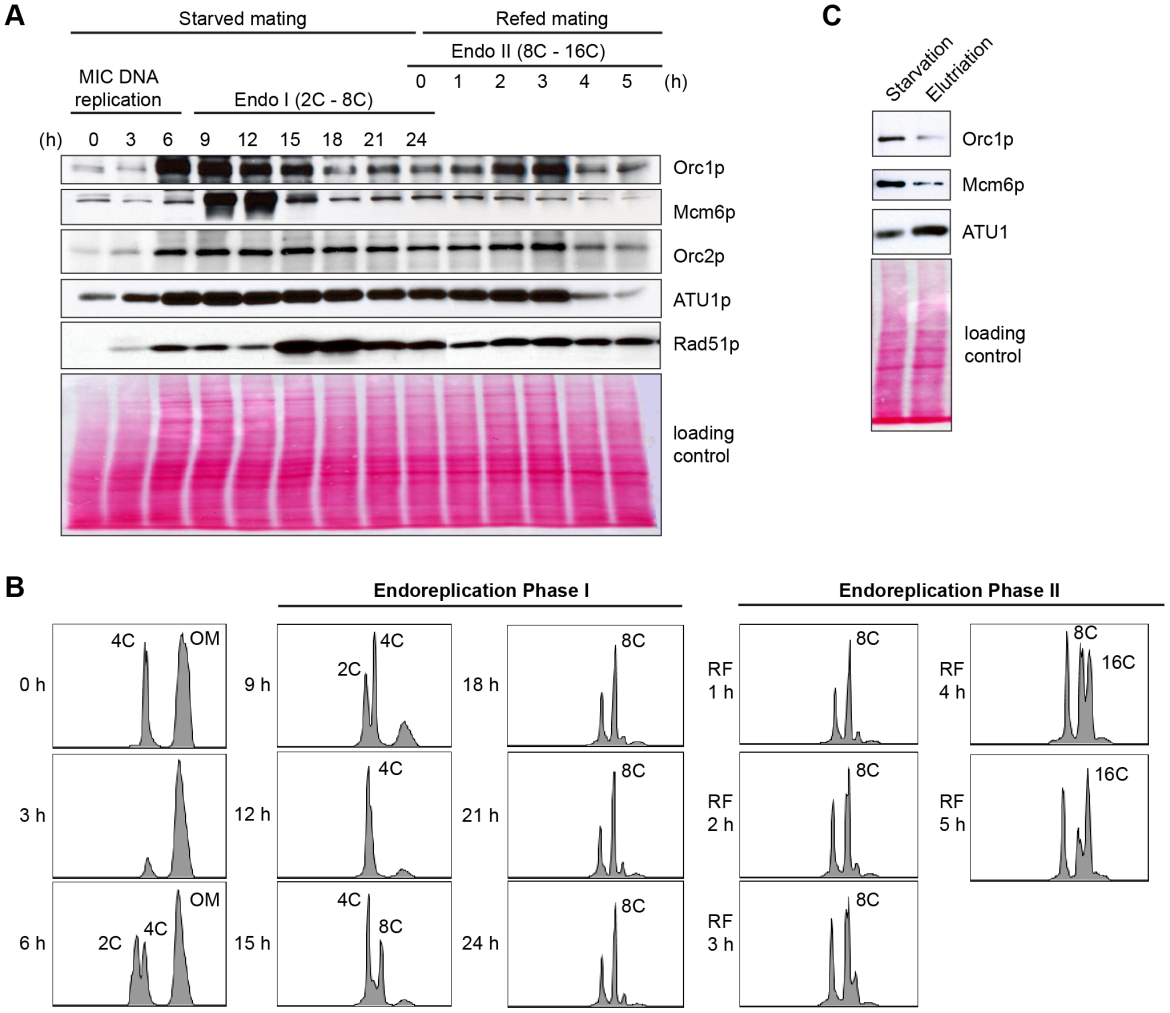
Developmental regulation of pre-RC components. Whole cell lysates were prepared from matings between wild type strains, CU427 and CU428, at indicated time points during conjugation. 0–6 h, micronuclear DNA replication; 9–24 h, endoreplication phase I (Endo I). Mating cultures were re-fed at 24 h to complete development (endoreplication phase II; Endo II). Equivalent amounts of total protein (20 µg) were separated by denaturing polyacrylamide gel electrophoresis and subjected to western blot analysis. (B) Flow cytometry analysis samples analyzed in panel A. Nuclei were isolated and stained with propidium iodide. Each histogram represents the number of counted nuclei (x-axis) versus DNA content (y-axis). OM, old parental macronucleus, which is degraded in conjugants. (C) Western blot analysis of wild type cells (CU428) synchronized at the G1/S border by starvation for 18 h or by centrifugal elutriation of a log phase vegetative culture.

Endoreplication occurs in two temporally separable stages in the developing macronucleus, both of which require expression of the ASI2 gene [35]. The first period (endoreplication phase I) involves two rounds of DNA replication and generates an 8 C macronucleus (Fig. 5A). In matings between wild type strains, the first increase in DNA content occurs between 6–18 h (Fig. 6B). Upon re-feeding, a second re-replication period (endoreplication phase II) is initiated in an ASI2-dependent manner [35], generating a macronuclear DNA content of 32–64 C (Fig. 6B, S4B Fig.). Endoreplication phase II was delayed in mating between ORC1-KD and a wild type partner (CU427), and was arrested in crosses with the wild type strain SB1934 (Fig. 5A). The parental/old macronucleus (OM) was not destroyed in ORC1 mutant x SB1934 progeny (Fig. 5A), further evidence for arrest of the developmental program. DAPI images of mating cells corroborated these findings (Fig. 5B). In wild type mating cultures (24 h mating, mated and re-fed for 4 and 8 h), the vast majority of exconjugant progeny exhibited robust DAPI staining of the two macronuclear anlagen (SB1934 x CU428). In contrast, the macronuclear DAPI signal was very faint in progeny from crosses with the ORC1 mutant.

**Figure 7:**
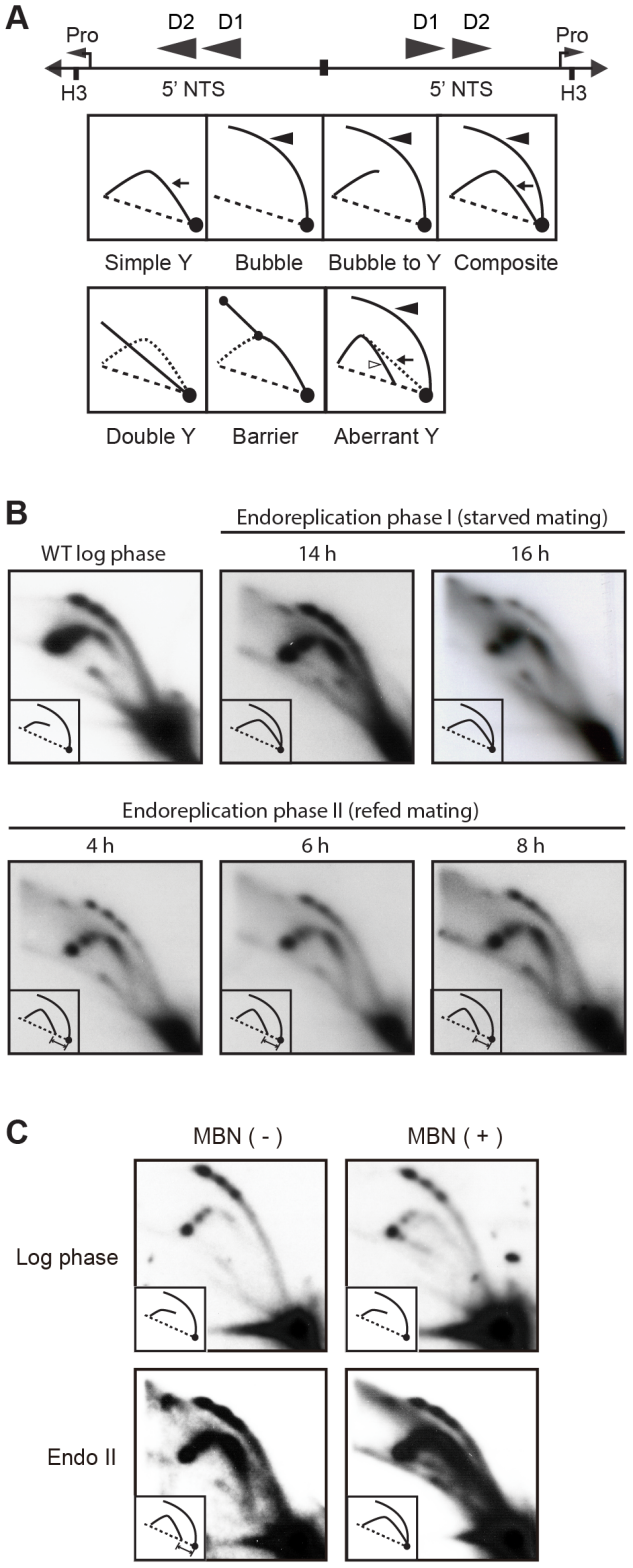
Two-dimensional gel analysis of rDNA replication intermediates during development. (A) Schematic of the palindromic rDNA 5′NTS fragment generated by *HindIII* (H3) digestion, and possible replication intermediate patterns resolved by neutral-neutral 2D gel electrophoresis. Pro, promoter; D1 and D2, imperfect 430 bp tandem duplications and harbor the rDNA origins of replication. Simple Y (arrow), passive replication of 5′ NTS origins. Bubble (filled arrowhead) or bubble to Y, bidirectional replication within the 5′ NTS. Composite (simple Y arc and bubbles), active and passive replication of the 5′ NTS. Double Y, two converging forks initiating within or outside the 5′ NTS. Barrier, replication of the 5′ NTS by converging forks, in which the first fork entered and terminated at a barrier prior to entry of the second fork. Aberrant Y (unfilled arrowhead), simple Y arc containing partially replicated DNA. Diagonal dashed line, migration of linear duplex DNA fragments; dotted arc, reference pattern for simple Y arc intermediates. (B) C3 rDNA amplification replication intermediates after mating for 14 and 16 h, and subsequent refeeding (at 24 h) for an additional 8 h. Mating: strains SB4202 and SB1934. (C) Mung bean nuclease (MBN) digestion on C3 rDNA amplification intermediates in matings between SB4202 and SB1934 collected after refeeding with media for 8 h. DNA samples from log phase SB4204 stain were collected as the control.

To determine if depletion of Orc1p affected rDNA amplification, Southern blot analysis was used to assess C3 rDNA production in a mating between the ORC1 mutant and wild type C3 rDNA strain, SB1934 [36]. The SB1934 heterokaryon contains two copies of the integrated C3 rDNA locus in its micronucleus and ∼9000 copies of the B rDNA allele in the macronucleus. Since the ORC1 knockdown strain encodes B rDNA in both nuclei, exconjugant progeny will contain a mixture of B and C3 rDNA in the developing macronucleus. In a cross between SB1934 and wild type B rDNA strain, CU428, C3 rDNA was amplified during macronuclear development (Fig. 5C). In contrast, C3 rDNA was not detected in progeny from the ORC1-KD x SB1934 cross. The collective data demonstrate that the experimentally induced down regulation of ORC1 inhibits global DNA replication and rDNA gene amplification during macronuclear development.

### Developmental regulation of ORC and MCMs subunits

Starvation not only induces cell cycle arrest at the macronuclear G1/S border, when Orc1 protein levels are highest during the vegetative cell cycle [23], it prepares cells for conjugation. The earliest rounds of DNA replication in this developmental program are restricted to micronucleus, while the later rounds create a new polyploid macronucleus. Published microarray profiles revealed two increases in the abundance of ORC and MCM subunit transcripts, peaking 4 h and 14 h after mixing starved cells with different mating types (S3 Fig. and S2 Table; http://tfgd.ihb.ac.cn/) [24], [37]. The more prominent early peak is derived from the parental macronucleus, suggesting that these proteins might be stockpiled for later use in the developing macronucleus.

To assess the abundance of pre-RC components throughout development, western blot analysis was performed with peptide antibodies specific for Tetrahymena Orc1p, Orc2p and Mcm6p. Alpha tubulin (Atu1p) and Rad51p were used as reference proteins for comparative analysis. RAD51 generates a distinct RNA profile during development in starved mating cells [38], while Atu1p levels have been reported to fluctuate minimally in starved mating cells [39]. Lowry assays were performed to assure that equivalent amounts of protein were loaded in each lane, and Ponceau S staining was used to monitor protein transfer prior to western blot probing.

Consistent with its RNA profile, Orc1p levels increased dramatically within the synchronized mating cell population, generating a broad peak between 6–15 h (Fig. 6A). While these time points encompass multiple rounds of micronuclear DNA replication and two rounds of macronuclear anlagen DNA replication (endoreplication phase I) (Fig. 6B), the cumulative amount of DNA replication is less than half the amount generated during a single vegetative cell cycle. Hence, ORC is in vast excess compared to the vegetative cell cycle. Centrifugal elutriation of a vegetative G1 phase population verified that starvation per se does not appreciably affect the abundance of Orc1p (Fig. 6C). The decline in Orc1p and Mcm6p levels prior to conjugant re-feeding (18–24 h) indicates that these proteins are not stockpiled for later use. A second wave of Orc1p synthesis was detected in mated re-fed cells. This occurred after the parental macronucleus was destroyed and prior to endoreplication phase II (Fig. 6A and 6B). Similar to Orc1p, Mcm6p levels rose and declined early in development (9–12 h). However, Mcm6p levels did not increase in mated/re-fed cells (endoreplication phase II). Like Atu1p, Orc2p were relatively constant throughout development. Notably, Rad51p levels peaked when Orc1p levels were low (i.e. endo I (15–18h), endo II 4–5 h) ruling out the possibility that the observed changes in Orc1p abundance can be generalized to all proteins, regardless of their function.

### Altered DNA replication during endoreplication phase II

The decline in Orc1p and Mcm6p at later time points during endoreplication phase II (Fig. 6A, re-fed 4 h and 5 h) prompted us to examine rDNA replication intermediates during this phase. To do so, two heterokaryon strains with a C3 rDNA micronucleus and B rDNA macronucleus were mated and RIs were examined by 2D gel electrophoresis. Western blot analysis and flow cytometry verified the reproducibility for the transient increase and subsequent decline in Orc1p in mated/re-fed cells, and revealed Orc1p levels did not further oscillate prior to the next round of endoreplication (16 C to 32 C) (S4A-S4B Fig.).

To visualize RIs generated in the developing macronucleus, newly synthesized macronuclear C3 rDNA molecules were resolved from B rDNA molecules (derived from the parental macronucleus and non-mating cells within the population) by treating *HindIII*-digested genomic DNA with *SphI*, which cleaves the B rDNA 5′ NTS into two smaller fragments. As previously reported [40], the predominant RI pattern detected in the 14–16 h window in mating cells is bubble-to-Y arc (Fig. 7A, schematic), which is generated from initiation events in one 5′ NTS copy in the rDNA palindrome, followed by the transient pausing of replication forks that migrate toward the telomere (Fig. 7B) [32]. This pattern is exclusively observed during vegetative S phase (Fig. 7B, WT log phase).

The rDNA replication pattern during endoreplication phase II displayed the general features of RIs in mated starved cells (endoreplication phase I) with the following difference: aberrantly migrating RIs were detected on the Y arc during endoreplication phase II (Fig. 7B, 4–8 h refed mating). Whereas the trajectory of low molecular weight Y arc intermediates in log phase and endoreplication phase I cells intersects the 1N (unreplicated) DNA spot, the trajectory of low molecular weight RIs generated during endoreplication phase II was shifted significantly to the left. Similar aberrant RIs accumulate in *S. cerevisiae* senataxin mutants, which are deficient in an RNA helicase that serves multiple roles in RNA metabolism [41]. Like senataxin mutants, the aberrant RIs, termed gapped forks, were eliminated when Tetrahymena DNA samples were sequentially treated with RNAse A and Mung Bean nuclease (MBN) (Fig. 7C). Most notably, the aberrantly migrating RIs are converted to simple Y arcs following MBN-treatment, consistent with the passive replication of the 5′ NTS origins. We speculate that these replication origins are not used in a sub-population of endoreplicating rDNA molecules, when ORC protein levels are lowest.

## Discussion

A distinguishing feature of the early branching eukaryotic phylum, Ciliophora, is the co-habitation of two functionally distinct nuclei within a single cytoplasm. Chromosomes in the somatic macronucleus are actively transcribed and contain euchromatic histone modifications that facilitate this process (i.e. histone H3K29Ac) [42]. These same DNA sequences (including replication origins) are packaged into heterochromatin in the transcriptionally silent germ line micronucleus. Whereas chromosomes segregate randomly in the polyploid macronucleus, DNA copy number is maintained through the elimination of excess DNA in the form of chromatin extrusion bodies [43] or re-replication of the genome when a minimal DNA content is not achieved [17]. Despite the imprecision of amitosis, macronuclear DNA replication is governed by the same regulatory mechanisms that function in canonical (G1-S-G2-M) cell cycles [44], [45]. They include ORC-dependent, site-specific initiation of DNA replication [22], [23], cell cycle regulated pre-RC assembly [23], S phase inactivation of ORC (to prevent re-replication) [23], [46], and the presence of a robust ATR-mediated DNA damage/replication stress checkpoint response [26].

In this study we examined the effect of modulating ORC1 on the execution of micro- and macronuclear DNA replication programs. The moderate reduction in ORC protein levels that was achieved compromised the integrity of chromosomes in micro- and macronuclei during the vegetative cell cycle, yet the deficiencies were not sufficient to trigger a robust intra-S phase checkpoint response. Remarkably, much greater fluctuations in ORC abundance occur during wild type Tetrahymena development. The successive changes in ORC and MCMs protein levels that we uncovered indicate that developmentally regulated replication programs are more complex than previously imagined.

### ORC, MCMs and the vegetative cell cycle

The compartmentalization of germ line and somatic functions into micro- and macronuclei poses unique challenges to master regulators of the cell cycle. Micro- and macronuclear S phases are offset during the vegetative cell cycle, daughter chromosomes are partitioned by radically different mechanisms (mitosis and amitosis), and mitotic nuclear division is not coupled to cytokinesis. Indeed, although macronuclear S phase precedes micronuclear S, the order of nuclear division is reversed. We previously showed that ORC (Orc1p, Orc2p and the integral Tetrahymena-specific RNA subunit, 26T RNA) dissociates from macronuclear replication origins during S phase, concomitant with the degradation of Orc1p. ORC is subsequently distributed randomly onto daughter chromosomes during G2 phase and re-localizes to origins in G1 phase [23]. To better illuminate the role(s) of ORC in replication origin licensing, we disrupted the ORC1 gene in the polyploid (45 C) macronucleus. As predicted for an essential gene, complete replacement was not achieved. The 5-fold decrease in ORC1 gene dosage was accompanied by a comparable reduction in Orc1p (Fig. 1B). An unexpected drop in Orc2p and Mcm6p levels was also observed in the ORC1 knockdown strain (Fig. 1B). This may reflect the co-regulation of pre-RC components (see below).

ORC1 mutants exhibited an elongated cell cycle that was typically accompanied by aberrant macronuclear division with lagging chromosomes (Fig. 1; S1 Fig.). The high incidence of abnormal cell division is consistent with the failure to activate the ATR checkpoint response. In support of this model, HU and MMS did not arrest cell cycle progression or inhibit nascent strand synthesis in the mutant (Fig. 2A and E), and the induction of Rad51p, a marker for checkpoint activation, was reduced (Fig. 2B–D). The essential role for ORC in the germ line micronucleus was highlighted by the instability of mitotic chromosomes during vegetative propagation of the mutant. The progressive loss of micronucleus-specific chromosome markers (Fig. 4A) led to defects in meiotic chromosome transmission (Fig. 4B) and sterility. Micronuclear chromosome loss failed to trigger cell cycle arrest during the vegetative cell cycle, consistent with previous reports for strains lacking the checkpoint activator protein, TIF1p, or harboring a fragile site in the micronuclear genome [26], [34]. We conclude that a moderate reduction in Orc1p levels profoundly compromises DNA replication in the micro- and macronucleus.

The simplest explanation for these phenotypes is that the reduction in Orc1p compromises replication initiation. Three lines of evidence argue against this model. First, two-dimensional gel analysis showed that rDNA origin site selection and/or usage were unaffected in the ORC1 knockdown strain (Fig. 3A). Second, DNA fiber imaging of non-rDNA chromosomes revealed no increase in the average inter-origin distance. Third, DNA fiber imaging uncovered a decrease in the rate of replication fork elongation (Fig. 3B). We postulate that the slowing of replication forks is linked to the down regulation of MCMs in the ORC1 mutant (Fig. 1B). Accordingly, the diminished DNA replication checkpoint response may reflect the propensity to maintain proper coupling between the replicative helicase and polymerase. This fork elongation defect is surprising for several reasons. Unlike Tetrahymena, down-regulation of *S. cerevisiae* ORC2 has no effect on MCM protein abundance and/or replication fork progression. Instead, origin utilization declines and the inter-origin distance increases in the ORC2-1 mutant strain [4]. Furthermore, MCMs are in excess to ORC in other model systems [47], [48]. This stoichiometry has been proposed to facilitate the initial unwinding of replication origins. Furthermore, excess MCMs have been shown to protect chromosomes from replication stress (i.e. stalled or collapsed forks) by activating neighboring dormant origins [49], [50]. These safeguards may not be necessary in the polyploid amitotic macronucleus of Tetrahymena, which employs other correcting mechanisms to maintain genic balance [17], [43]. Finally, partial depletion of MCMs preferentially inhibits replication initiation in *S. cerevisiae* and cultured Drosophila cells [51], [52], suggesting that this step is most sensitive to MCM dosage. The down regulation of Mcm6p in Tetrahymena ORC1 mutants and accompanying effect on replication fork progression argue that elongation is the rate limiting step when ORC and MCM levels are reduced in this species. Whether this reflects how origins are distributed throughout the Tetrahymena genome (i.e. dispersed versus clustered origins [53]), different requirements for ORC:MCM stoichiometry, or alternative mechanisms for replication initiation in the amitotic macronucleus awaits further studies.

### ORC, MCMs and development

In contrast to the vegetative cell cycle, micro- and macronuclear DNA replication programs are uncoupled during Tetrahymena development. The increased production of maternal transcripts encoding replication pathway proteins (S3 Fig.) [24] suggested that these factors might be stockpiled for later use in progeny, when the demands for DNA replication increase. The earliest rounds of DNA replication in conjugating cells are exclusively devoted to the micronuclear genome. They generate stationary and migratory pronuclei for reciprocal genetic exchange, and produce four genetically equivalent micronuclei in exconjugants. Subsequent rounds of DNA replication are restricted to the macronuclear ‘anlagen’, as nonconventional DNA replication programs (endoreplication, rDNA gene amplification) are activated.

The developmental oscillations in ORC and MCM protein levels that we uncovered suggest that the rules for DNA replication change at different stages of development. They are intriguing because ORC levels do not correlate with the amount of DNA that is synthesized at a given time. Orc1p levels are highest early in development, when the micronucleus alone is being replicated (Fig. 6). They start to decline during early rounds of endoreplication in the developing anlagen (4–8 C). Conversely, these proteins are at their lowest level when the demands for macronuclear DNA synthesis peak in late stage endocycling cells (Fig. 6, S4 Fig., 16–32 C).

While the purpose for the initial increase in Tetrahymena ORC and MCM abundance awaits further investigation, it appears to be distinct from the replication programs associated with early embryonic development in Xenopus and Drosophila [10], [54]. In all three cases, maternally derived proteins support DNA replication prior to cell differentiation. In flies and frogs, ORC and MCM levels are elevated until the mid-blastula transition. They correlate with a transient increase in origin density and occur in transcriptionally silent nuclei. ORC and MCM levels plummet following the onset of zygotic transcription, and subsequent initiation events are primarily relegated to intergenic chromosomal regions. The functional connection between transcriptional silencing and elevated ORC does not hold for Tetrahymena. The micronucleus is transcriptionally quiescent during the vegetative stage of the life cycle, and is actively transcribed during development, generating non-coding RNAs that direct the removal of micronuclear-limited sequences in macronuclear anlagen [18], [55]. Furthermore, the demands for DNA replication during Tetrahymena development are markedly reduced relative to metazoa. In the case of Drosophila, successive ten minute cell cycles generate ∼6000 nuclei in the developing syncytium. Tetrahymena generates only four diploid micronuclei prior to the onset of zygotic transcription. The net increase in synthesized DNA is only 6 C.

Whereas the primary goals of endoreplication phases I and II are the same, to increase DNA content in the newly developing progeny macronucleus, these replication programs have several distinguishing characteristics. Both programs require the ASI2 gene, which encodes a putative transmembrane protein that may function in signal transduction [35], [56], analogous to Notch-dependent signaling in endocycling Drosophila follicle cells [57]. However, unique extrinsic and/or intrinsic requirements must exist, since endoreplication phase I occurs in starved mating cells and endoreplication phase II requires re-feeding. Furthermore, rDNA gene amplification is restricted to endoreplication phase I, and occurs concurrently with the replication of non-rDNA chromosomes [36], [40]. Finally, ORC levels are reduced during endoreplication phase II (Fig. 6 and S4 Fig.), and unconventional DNA replication intermediates are produced at this time (Fig. 7), indicative of an altered DNA replication program (see below).

Our studies with the ORC1 knockdown strain clearly demonstrate that ORC is required for endoreplication and rDNA gene amplification (Fig. 5). While the contributions of ORC to endoreplication phase I are well supported, the dependence of ORC during endoreplication phase II is less clear. For example, the rDNA is exclusively replicated from 5′ NTS origins during endoreplication phase I (Fig. 7; bubble-to-Y arc, no simple Y arcs). Endoreplication phase II is much more complicated. While a bubble-to-Y arc pattern is seen, consistent with ORC-mediated initiation, a new pattern of aberrantly migrating ‘Y-like’ RIs is also observed. The sensitivity of the Y-like RIs to sequential ribonuclease-A (RNase) and Mung Bean nuclease (MBN) treatment supports the idea that single strand DNA or stable RNA-DNA hybrids accumulate in the 5′ NTS region during endoreplication phase II. Their nucleolytic conversion to simple Y arc RIs argues that the initiation site is not coincident with the known, ORC-dependent replication origins. By analogy, the impaired activity of an *S. cerevisiae* RNA helicase, senataxin leads to the accumulation of RNA-DNA hybrids, and generates RNase/MBN-sensitive RIs [41]. Moreover, aberrant RIs accumulate when Orc1p levels decline in the developing macronuclear anlagen (S4 Fig.). Since the experimental down regulation of ORC in vegetative cells does not produce this aberrant pattern (Fig. 3), their genesis may be restricted to Tetrahymena development. We propose that the rRNA promoter or a cryptic 5′ NTS promoter generates RNA-DNA hybrids in endocycling Tetrahymena, and speculate that this RNA might inhibit initiation from the ORC-dependent origin or serve as a primer for DNA synthesis.

How might replication initiation be achieved with limiting amounts of ORC? It is conceivable that ORC might transiently associate with origins to establish pre-RCs and dissociate once MCM complexes are loaded. Live imaging recently revealed that ORC and CDC6 turn over rapidly on chromatin in *C. elegans* embryos, but MCMs do not [58]. Alternatively, replication initiation and elongation might be temporally uncoupled during endoreplication phase II, as was reported for amplification of the chorion gene locus in stage 10–13 Drosophila embryos [59]. Our previous data studies of the vegetative cell cycle do not support either model in Tetrahymena. ChIP analysis of synchronized cell populations demonstrated that ORC is randomly deposited onto chromatin during G2 phase and re-localizes to replication origins during G1 phase, concomitant with the recruitment of MCM complexes [23]. Fundamental changes in ORC-dependent pre-RC assembly would be required to support endoreplication with diminished amounts of ORC. Furthermore, the aberrant RIs that form during endoreplication phase II are inconsistent with re-initiation of DNA replication on stalled replication forks (onion skin replication). Indeed, onion skin RIs accumulate very early in the amplification process due to the activation of a developmentally programmed replication fork barrier [40].

A more provocative possibility is that endoreplication occurs by an ORC-independent mechanism. Although ORC normally associates with chromatin in endocycling Drosophila salivary gland cells [60], the ability of ORC2 mutant clones to support genome-wide re-replication is consistent with this model [61]. Furthermore, origin-independent propagation of chromosomes in *S. cerevisiae* [62] and *Haloferax volcanii* [63] demonstrates that recombination-based mechanisms can propagate chromosomes when ORC-mediated pre-RC assembly is perturbed. Advances in genome-wide analysis, such as nascent strand-seq [64] should provide fundamental insights in the underlying mechanism for ‘alternative’ DNA replication programs in Tetrahymena.

## Materials and Methods

### DNA transformation and propagation of *Tetrahymena thermophila* strains

Tetrahymena strains are described in S1 Table. Standard methods were used for mating, transfection and selection of paromomycin (pm) resistant transformants, and for vegetative propagation of wild type and mutant *Tetrahymena* strains [26]. The ORC1 knockdown strain (TD101) was generated by replacing the ORC1 protein coding region with an MTT1-neo gene cassette, using flanking ORC1 sequences for targeted homologous recombination [23]. Bio-ballistic transformation was used to introduce DNA into the developing macronucleus in a mating between strains CU427 and CU428. Paromomycin (pm) resistant progeny were continuously selected for ‘phenotypic assortants’ with increased levels of drug resistance (100 µg/ml to 1000 µg/ml), due to replacement of wild type copies of the ORC1 gene with an MTT1-neo disruption allele in the polyploid amitotic macronucleus.

### Cell cycle synchronization and flow cytometry

Cell cycle synchronization was achieved by starvation and re-feeding or centrifugal elutriation as previously described [23]. Flow cytometric analysis was performed to monitor cell cycle progression in vegetative growing cells [25], and to determine the relative DNA content in isolated micro- and macronuclear populations in mating cells. For the later, nuclei were extracted in ice-cold nucleus extraction buffer (10 mM Tris at pH 7.4, 10 mM MgCl_2_, 3 mM CaCl_2_, 0.25 M sucrose, 0.2% NP-40, 1 mM dithiothreitol, 1 mM phenylmethylsulfonyl fluoride). Flow cytometry analysis was performed on Becton Dickinson FACSAria II flow cytometer (BD Biosciences). Data were analyzed using BD FACSDiva software. Histograms were plotted, in which the y-axis represents the number of events and the x-axis represents the relative DNA content.

### Cell fractionation and western blot analysis

Whole cell extracts were prepared by lysing cells in 1% SDS lysis buffer (50 mM Tris at pH 8.0, 150 mM NaCl, 1 mM EDTA, 1% NP-40, 1% sodium deoxycholate, 1% SDS) for 15 min on ice. To prepare soluble and chromatin-bound fractions, cells were incubated in 50 µl NP-40 lysis buffer (0.1% NP-40, 20 mM Tris-HCl at pH 8.0, 137 mM NaCl, 10% glycerol, 2 mM EDTA) for 10 min on ice. After centrifugation at 2,000 rpm for 10 min at 4°C, the supernatant was collected as the ‘soluble fraction’. The pellet was resuspended in 50 µl of 1% SDS lysis buffer and designated as ‘chromatin-bound fraction’. Protein concentrations were determined using the modified Lowry protein assay reagents (Bio-Rad, cat# 500-0111). Unless otherwise stated, equal amounts of total protein (20 µg) were loaded in each lane and samples were subjected to SDS-PAGE and transferred to nitrocellulose membranes (Whatman Protran BA85, GE Healthcare). Prior to probing, membranes were stained with Ponseau S staining (Sigma-Aldrich) to confirm equivalent sample loading and protein transfer. Immunodetection of Tetrahymena Orc1p (1∶5,000), Orc2 (1∶5,000) and Mcm6p (1∶10,000) were carried out using polyclonal rabbit antibodies raised against immunogenic Tetrahymena peptides (Covance). Antibodies directed against Rad51 (51RAD01, Thermo Scientific, 1∶5,000 dilution), acetyl-histone H3 (Upstate (Invitrogen), 1∶10,000 dilution), and alpha tubulin antibodies (Abcam, 1∶2,000 dilution) were obtained from the indicated commercial sources. Blots were incubated with the primary antibodies at 4°C overnight, washed and incubated for 3 h at 4°C with horseradish peroxidase-conjugated goat anti-rabbit or anti-mouse IgG (Jackson ImmunoResearch). Membrane bound secondary antibodies were visualized using ECL reagents (PerkinElmer) according to the manufacturer's instructions. Densitometry of blots was performed using ImageJ software (version 1.47v for Macintosh, National Institutes of Health).

### Cytological staining and fluorescence microscopy

For mating experiments, wild type strain CU427 was distinguished from its mating partner by incorporation of Mitotracker Red-CMXRos (Molecular Probes, Invitrogen) at a concentration of 500 nM during overnight starvation at 30°C. On the next day, starved cell cultures were washed once and resuspended in 10 mM Tris at the density of 2.5×10^5^ cells/ml. Mating was initiated by mixing CU427 with CU428 or CU427 with ORC1 knockdown cells in equal volume. One-milliliter of mating cultures were harvested each hour during development. Cells were fixed in 70% ethanol for at least 1 h. Fixed cells were washed once with PBS and resuspended in 100 µl of 0.1 µg/ml 4′,6′-diamidino-2- phenylidole (DAPI, Sigma Chemical) to stain nuclei. To visualize nuclear division, 1 ml of log phase CU427 and ORC1 knockdown cell cultures were harvested and fixed with 2% paraformaldehyde. Acridine orange was added at a concentration of 0.001% to stain nuclei. Cells were examined by conventional fluorescence microscopy.

### DNA isolation and enrichment for replication intermediates (RIs)

Total genomic DNA was isolated from Tetrahymena cultures as previously described to preserve DNA replication intermediates [40]. For RI enrichment, 200 µg of genomic DNA were digested with *HindIII* for 4 h and applied to the 200-μl packed volume benzoylated naphthoylated DEAE (BND)-cellulose (Sigma-Aldrich). Caffeine-eluted DNA samples were precipitated with isopropanol, using 20 µg glycogen as a carrier. Samples were subsequently digested with *SphI* to resolved C3 and B rDNA fragments as needed. Total DNA recovery was estimated to be ∼5% of the input. For mung bean nuclease (MBN) treatment, 3 µg of BND cellulose-enriched RIs was digested with 5 U of MBN (New England BioLabs) at 30°C for 30 min. SDS was added to the final concentration of 0.01% to inactivate MBN [41].

### Two-dimensional (2D) gel electrophoresis of DNA replication intermediates

Neutral-neutral 2D gel electrophoresis was performed as previously described [40]. Approximately 3–10 µg of BND cellulose-enriched DNA was loaded for each 2D gel experiment. The first dimension gel (0.4% agarose) was run in 1X TAE buffer (40 mM Tris, 20 mM acetic acid, and 1 mM EDTA) at 1.5 V/cm for 20 h at RT. The second dimension gel (1% agarose) was run in 1X TBE buffer (90 mM Tris, 90 mM Boric acid, 2 mM EDTA) containing 0.5 mg/ml ethidium bromide at 3 V/cm for 18 h at 4°C. DNA was transferred overnight to a charged nylon membrane (Hybond-XL, Amersham) in alkaline buffer by capillary blotting. Membranes were prehybridized at 37°C for 4 h in 1M NaCl, 1% SDS, 10% dextran sulfate, 5 mM Tris [pH 7.5], 100 µg/ml of denatured salmon sperm DNA and 25% formamide. rDNA 5′NTS probes were labeled by random priming and added directly to the prehybridization solution. After 18 h membranes washed 3 times in 2X SSC/1% SDS solution for 15 min each at 42°C, and once in 0.4X SSC/0.1% SDS solution for 15 min at 42°C. Blot were exposed to X-ray film with an intensifying screen at −70°C or analyzed with a phosphorimager.

### Alkaline gel electrophoresis

Alkaline gel electrophoresis was performed to examine the replication initiation and elongation under HU treatment. Wild type CU428 and ORC1 knockdown cells were starved overnight to synchronize in G1 phase, and then released into medium containing 20 mM HU. Genomic DNA was isolated by phenol-chloroform extraction as described above from cells harvested at indicated time points under HU treatment. Sixty μg of genomic DNA was loaded in a 1% alkaline gel (40 mM NaOH, 2 mM EDTA at pH 8.0) and separated by electrophoresis in the alkaline buffer (40 mM NaOH, 2 mM EDTA) at 1.5 V/cm for 20 h at 4°C with buffer recirculation to resolve nascent-strand replication intermediates. After electrophoresis, DNA was transferred to a charged nylon membrane (Hybond-XL, Amersham) by capillary blotting and hybridized to the rDNA 5′NTS fragment that was radiolabeled with [α-^32^P]-dATP as described above.

### DNA fiber analysis

Tetrahymena strains CU428 and ORC1-KD were cultured in 2% PPYS media to the density of 1.5×10^5^ cell/ml. Cells were pulse labeled with 400 µM IdU (Sigma) at 30°C for 10 min. The media was removed and cells were washed once with 1X PBS. Cells were resuspended in pre-warmed fresh media with 100 µM CldU (MP Biomedicals) and labeled for 10 min. After two washes with PBS, the cell density was adjusted to ×10^6^ cells/ml. Preparation and immunostaining of DNA fibers was performed as described previously described [65]–[67] with the following modifications. Briefly, after fixation and HCl treatment, slides were washed three times with 1X PBS, and blocked with 5% BSA in PBS for 30 min. Mouse anti-BrdU (1∶50, Becton Dickson) and rat anti-BrdU (1∶100, Accurate Chemical) antibodies in 5% BSA were then added onto slides. After 1 h incubation, the slides wash washed three times again with 1X PBS, and incubated for 30 min with secondary antibodies: Alexa Fluor 568 goat anti-mouse IgG (1∶100, Invitrogen/Molecular probes) and Alexa Fluor 488 goat anti-rat IgG (1∶100, Invitrogen/Molecular Probes). Finally, slides were washed three times with 1X PBS, dehydrated with an ethanol series, and mounted with SlowFade Gold antifade solution (Invitrogen). During immunostaining, all antibodies were diluted in 5% BSA in 1X PBS, all incubations were performed at 37°C, and all wash steps were done at RT. DNA fiber images were taken by a Nikon A1R+ confocal microscope with 600X magnification. Measurements of track length were performed with Nikon NIS-Elements software. Inter-origin distance was defined as the distance between the centers of two red segments in either green-red-green-red-green or green-red-gap-red-green tracks. Fork velocity was determined by measuring the length of the green segment in a red-green track. GraphPad Prism software was used to analyze the statistical significance, and the p-values shown in figures were determined by two-tailed unpaired t-test.

## Supporting Information

S1 Fig Nuclear division in wild type CU428 and ORC1 knockdown cells visualized with acridine orange. Log phase wild type (WT) CU428 and ORC1 knockdown (ORC1-KD) cell cultures were collected and fixed with paraformaldehyde. For apofluor staining, cells were stained with 0.001% acridine orange and observed immediately with fluorescence microscopy. Eight representative images from each strain are shown.
(TIF)

S2 Fig Abrogated intra-S phase checkpoint response in ORC1 knockdown cells. Elutriated G1 phase wild type (CU428) and ORC1 knockdown (ORC1-KD) cells were treated with 0.06% MMS and samples were collected at the indicated intervals for flow cytometry analysis.
(TIF)

S3 Fig Microarray gene expression data of Tetrahymena pre-RC components. Gene expression profiles were accessed from Tetrahymena functional genomics database (TetraFGD) [24], [37]. Each profile contains 20 time points during the three physiological and developmental stages of the *T. thermophila* life cycle, including 3 points in growth (L), 7 points in starvation (S) and 10 points in conjugation (C). For growing cells, L-l, L-m and L-h correspond respectively to 1×10^5^ cells/ml, 3.5×10^5^ cells/ml and 1×10^6^ cells/ml. For starved cells, samples were collected at 0, 3, 6, 9, 12, 15 and 24 h (S0 – S24). For conjugation, samples were collected at 0, 2, 4, 6, 8, 10, 12, 14, 16 and 18 h after mating (C0 – C18). Blue and red lines represent the expression values normalized by two different methods. Tetrahymena ORC1 (Gene ID: TTHERM_00865050), ORC2 (Gene ID: TTHERM_00684560), MCM6 (Gene ID: TTHERM_00448570), CDT1 (Gene ID: TTHERM_00277530), PCNA (Gene ID: TTHERM_ 01107420), DNA polymerase epsilon, Β subunit (GENE ID: TTHERM_00614820), RAD51 (GENE ID: TTHERM_00142330), PDD1 (GENE ID: TTHERM_00125280).
(TIF)

S4 Fig Developmental regulation of pre-RC components. (A) Whole cell lysates were prepared from matings between wild type strains, CU427 and CU428, at indicated time points during conjugation. 0 h and 24 h: starved mating cells. Mated cells were re-fed at 24 h and samples were collected at 1 h interval for an additional 8 h. Equivalent amounts of total protein (20 µg) were separated by denaturing polyacrylamide gel electrophoresis and subjected to western blot analysis. (B) Flow cytometry analysis samples analyzed in panel A. Nuclei were isolated and stained with propidium iodide. Each histogram represents the number of counted nuclei (x-axis) versus DNA content (y-axis). OM, old parental macronucleus, which is degraded in conjugants.
(TIF)

S1 Table Genotypes and phenotypes of *T. thermophila* strains used in this study. The micronuclear alleles, chx1-1 and mpr1-1 confer resistance to cycloheximide and 6-methylpurine, respectively. C3 and B rDNA alleles can be distinguished by restriction fragment polymorphisms in the 5′ NTS or 3′ NTS. Homologous gene replacement of the wild type ORC1 gene with the ORC1:MTT-neo sequence confers resistance to paramomycin due to expression of the cadmium inducible neomycin phosphotransferase gene.
(DOCX)

S2 Table Coefficients for gene expression. The ORC1 gene expression profile was used as a reference to identify other DNA replication and repair genes with statistically significant coefficients for gene expression. Data were obtained from the TFGD database (http://tfgd.ihb.ac.cn/tool/network).
(DOCX)
