# Supplementary figures and images for: Developmental Regulation of the Tetrahymena thermophila Origin Recognition Complex
Source: PLoS Genet. 2015 Jan 8;11(1):e1004875. doi: 10.1371/journal.pgen.1004875 (PMC4287346; doi:10.1371/journal.pgen.1004875)

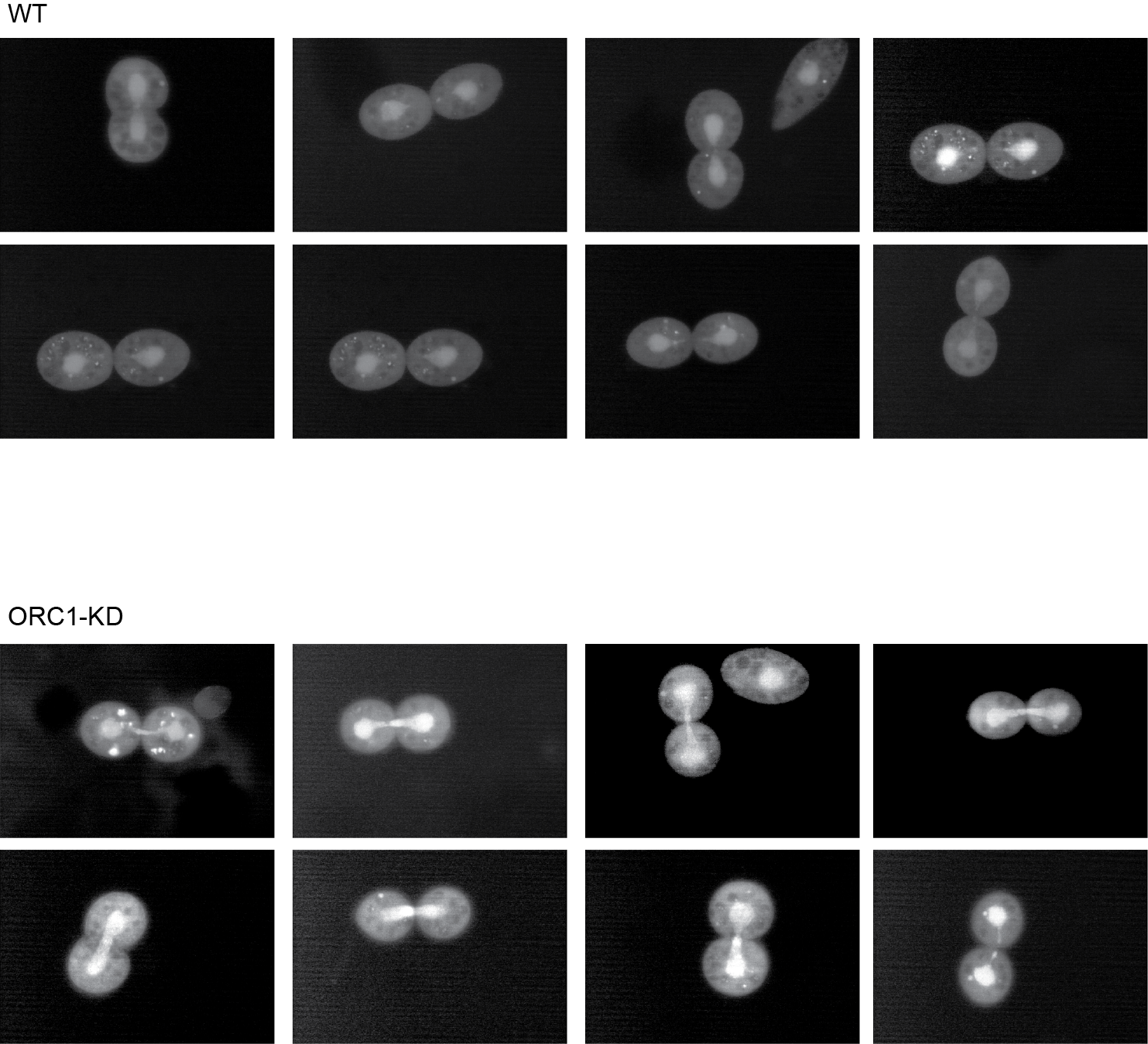

Supplement: S1 Fig — Nuclear division in wild type CU428 and ORC1 knockdown cells visualized with acridine orange. Log phase wild type (WT) CU428 and ORC1 knockdown (ORC1-KD) cell cultures were collected and fixed with paraformaldehyde. For apofluor staining, cells were stained with 0.001% acridine orange and observed immediately with fluorescence microscopy. Eight representative images from each strain are shown. (TIF) [file pgen.1004875.s001.tif]

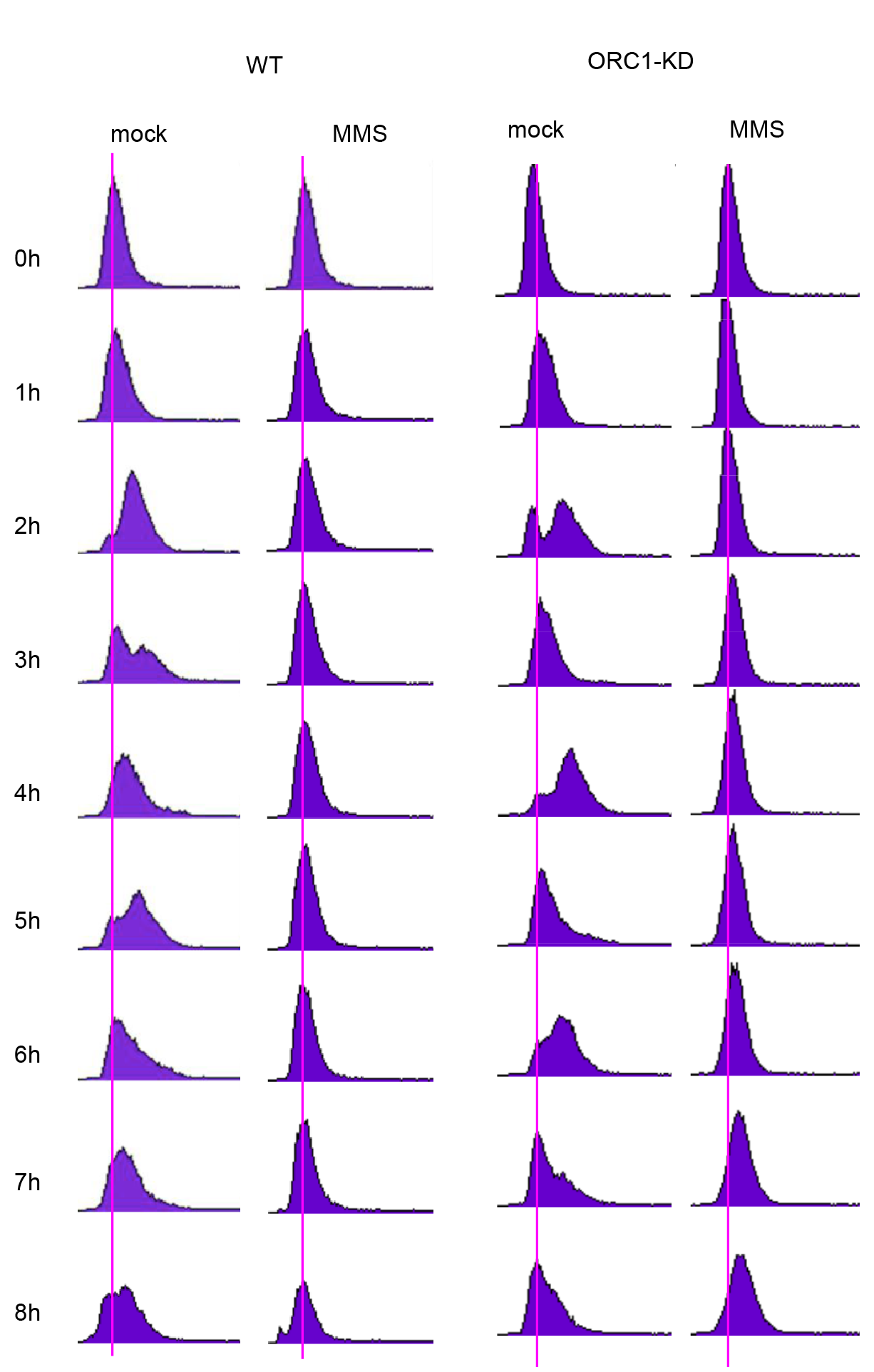

Supplement: S2 Fig — Abrogated intra-S phase checkpoint response in ORC1 knockdown cells. Elutriated G1 phase wild type (CU428) and ORC1 knockdown (ORC1-KD) cells were treated with 0.06% MMS and samples were collected at the indicated intervals for flow cytometry analysis. (TIF) [file pgen.1004875.s002.tif]

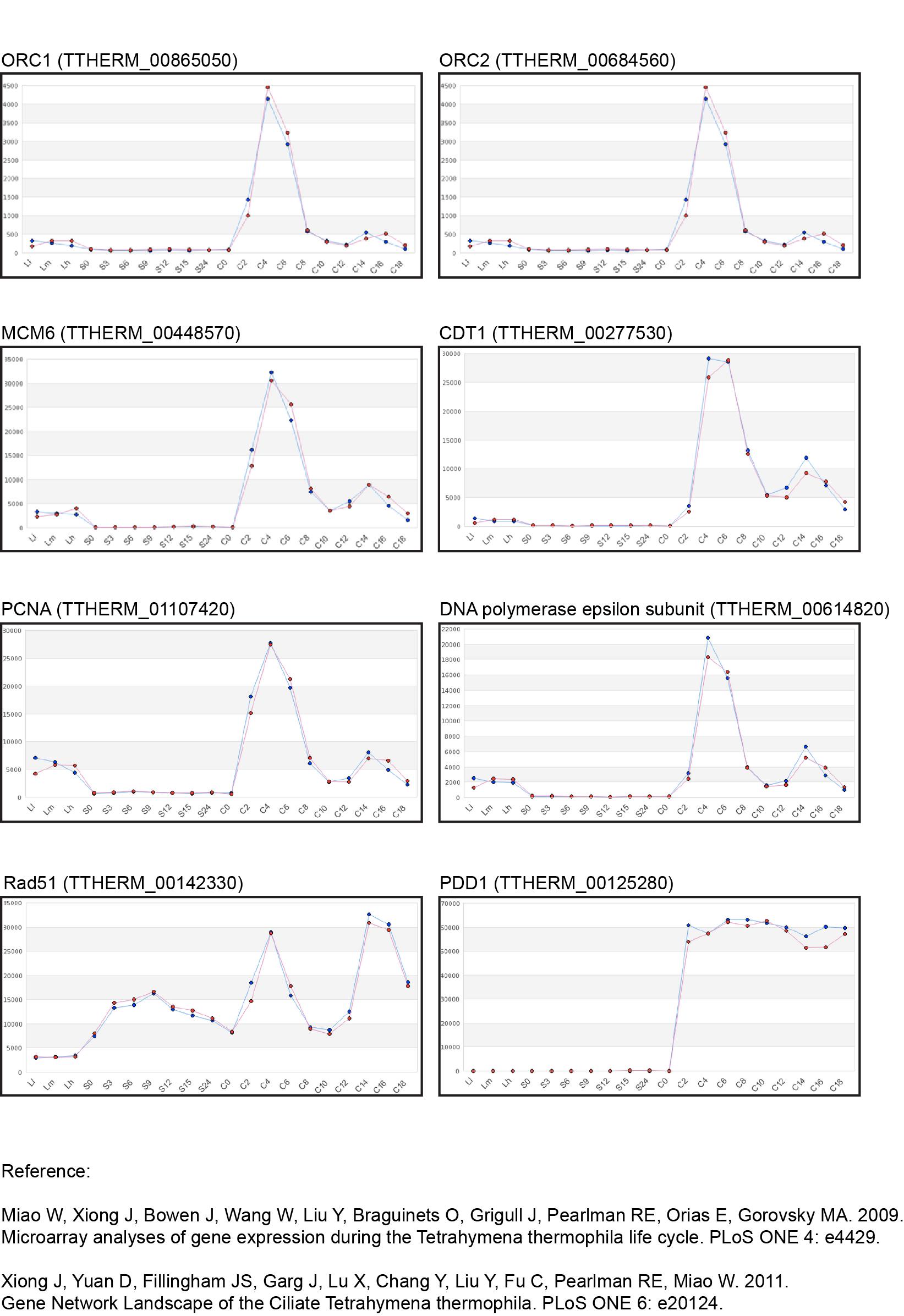

Supplement: S3 Fig — Microarray gene expression data of Tetrahymena pre-RC components. Gene expression profiles were accessed from Tetrahymena functional genomics database (TetraFGD) [24], [37]. Each profile contains 20 time points during the three physiological and developmental stages of the T. thermophila life cycle, including 3 points in growth (L), 7 points in starvation (S) and 10 points in conjugation (C). For growing cells, L-l, L-m and L-h correspond respectively to 1×10^5^ cells/ml, 3.5×10^5^ cells/ml and 1×10^6^ cells/ml. For starved cells, samples were collected at 0, 3, 6, 9, 12, 15 and 24 h (S0 – S24). For conjugation, samples were collected at 0, 2, 4, 6, 8, 10, 12, 14, 16 and 18 h after mating (C0 – C18). Blue and red lines represent the expression values normalized by two different methods. Tetrahymena ORC1 (Gene ID: TTHERM_00865050), ORC2 (Gene ID: TTHERM_00684560), MCM6 (Gene ID: TTHERM_00448570), CDT1 (Gene ID: TTHERM_00277530), PCNA (Gene ID: TTHERM_ 01107420), DNA polymerase epsilon, Β subunit (GENE ID: TTHERM_00614820), RAD51 (GENE ID: TTHERM_00142330), PDD1 (GENE ID: TTHERM_00125280). (TIF) [file pgen.1004875.s003.tif]

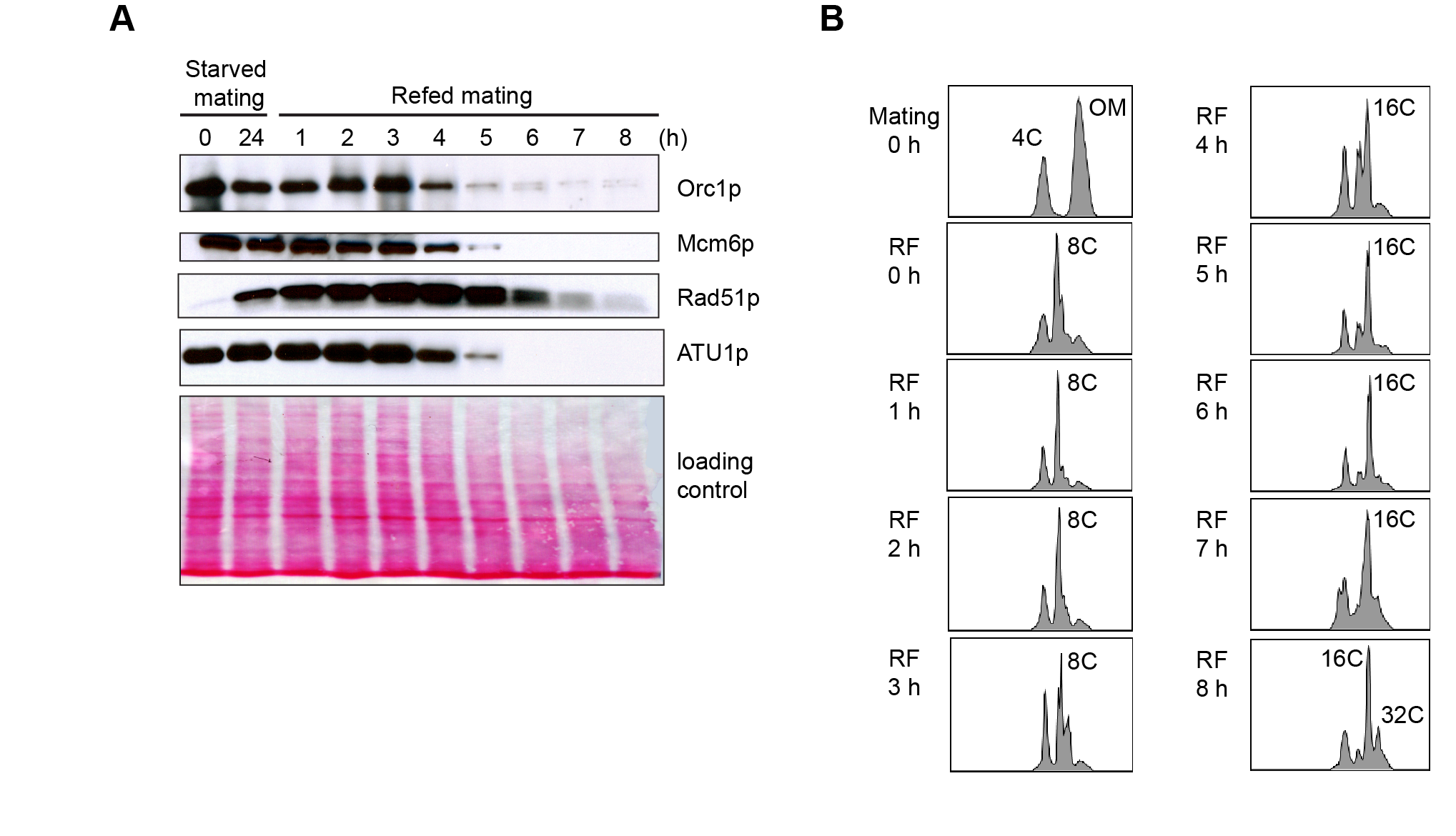

Supplement: S4 Fig — Developmental regulation of pre-RC components. (A) Whole cell lysates were prepared from matings between wild type strains, CU427 and CU428, at indicated time points during conjugation. 0 h and 24 h: starved mating cells. Mated cells were re-fed at 24 h and samples were collected at 1 h interval for an additional 8 h. Equivalent amounts of total protein (20 µg) were separated by denaturing polyacrylamide gel electrophoresis and subjected to western blot analysis. (B) Flow cytometry analysis samples analyzed in panel A. Nuclei were isolated and stained with propidium iodide. Each histogram represents the number of counted nuclei (x-axis) versus DNA content (y-axis). OM, old parental macronucleus, which is degraded in conjugants. (TIF) [file pgen.1004875.s004.tif]
