# Supplementary material for: Developmental Regulation of the Tetrahymena thermophila Origin Recognition Complex
Source: PLoS Genet. 2015 Jan 8;11(1):e1004875. doi: 10.1371/journal.pgen.1004875 (PMC4287346; doi:10.1371/journal.pgen.1004875)
Supplement: S1 Table — Genotypes and phenotypes of T. thermophila strains used in this study. The micronuclear alleles, chx1-1 and mpr1-1 confer resistance to cycloheximide and 6-methylpurine, respectively. C3 and B rDNA alleles can be distinguished by restriction fragment polymorphisms in the 5′ NTS or 3′ NTS. Homologous gene replacement of the wild type ORC1 gene with the ORC1:MTT-neo sequence confers resistance to paramomycin due to expression of the cadmium inducible neomycin phosphotransferase gene. (DOCX) [file pgen.1004875.s005.docx]

**Table S1.** *T. thermophila* strains used in this study.

| **Strain** | **Micronuclear genotype** | **Macronuclear phenotype** |
| --- | --- | --- |
| CU427 | *ORC1/ORC1*  *chx1-1/chx1-1* | paromomycin-sensitive  cycloheximide-sensitive |
| CU428 | *ORC1/ORC1*  *mpr1-1/mpr1-1* | paromomycin-sensitive  6-methylpurine-sensitive |
| ORC1-KD | *ORC1/ORC1*  *CHX1/chx1-1*  *MPR1/mpr1-1* | paromomycin-resistant; ORC1::MTT-neo*  cycloheximide-resistant  6-methylpurine-resistant |
| SB4202 | *ORC1/ORC1*  *rDNA [C3]/rDNA [C3]* | paromomycin-sensitive  rDNA [B] |
| SB1934 | *ORC1/ORC1*  *rDNA [C3]/rDNA [C3]* | paromomycin-sensitive  rDNA [B] |

* partial replacement
