# Supplementary material for: Developmental Regulation of the Tetrahymena thermophila Origin Recognition Complex
Source: PLoS Genet. 2015 Jan 8;11(1):e1004875. doi: 10.1371/journal.pgen.1004875 (PMC4287346; doi:10.1371/journal.pgen.1004875)
Supplement: S2 Table — Coefficients for gene expression. The ORC1 gene expression profile was used as a reference to identify other DNA replication and repair genes with statistically significant coefficients for gene expression. Data were obtained from the TFGD database (http://tfgd.ihb.ac.cn/tool/network). (DOCX) [file pgen.1004875.s006.docx]

**Table S2.**

| **Gene name** | **Gene Model Identifier** | **Z-score with ORC1** | **Link to TetraFGD** |
| --- | --- | --- | --- |
| ORC1 | TTHERM_00865050 | - | <http://tfgd.ihb.ac.cn/search/detail/gene/TTHERM_00865050> |
| ORC2 | TTHERM_00684560 | 6.96 | <http://tfgd.ihb.ac.cn/search/detail/gene/TTHERM_00684560> |
| MCM2 | [TTHERM_00554270](http://ciliate.org/index.php/feature/details/TTHERM_00554270) | 8.85 | <http://tfgd.ihb.ac.cn/search/detail/gene/TTHERM_00554270> |
| MCM3 | TTHERM_00092850 | 9.09 | <http://tfgd.ihb.ac.cn/search/detail/gene/TTHERM_00092850> |
| MCM4 | TTHERM_00277550 | 8.84 | <http://tfgd.ihb.ac.cn/search/detail/gene/TTHERM_00277550> |
| MCM5 | TTHERM_00069420 | 5.82 | <http://tfgd.ihb.ac.cn/search/detail/gene/TTHERM_00069420> |
| MCM6 | TTHERM_00448570 | 8.26 | <http://tfgd.ihb.ac.cn/search/detail/gene/TTHERM_00448570> |
| MCM7 | TTHERM_00011740 | 7.77 | <http://tfgd.ihb.ac.cn/search/detail/gene/TTHERM_00011740> |
| MCM8 | TTHERM_01031060 | NC | <http://tfgd.ihb.ac.cn/search/detail/gene/TTHERM_01031060> |
| MCM9 | TTHERM_00703910 | 4.43 | <http://tfgd.ihb.ac.cn/search/detail/gene/TTHERM_00703910> |
| PCNA | TTHERM_01107420 | 7.11 | <http://tfgd.ihb.ac.cn/search/detail/gene/TTHERM_01107420> |
| DNA polymerase alpha/primase | TTHERM_00424700 | 7.23 | <http://tfgd.ihb.ac.cn/search/detail/gene/TTHERM_00424700> |
| RFC1 | TTHERM_00939110 | 4.54 | <http://tfgd.ihb.ac.cn/search/detail/gene/TTHERM_00939110> |
| RFC2 | TTHERM_00245150 | 4.16 | <http://tfgd.ihb.ac.cn/search/detail/gene/TTHERM_00245150> |
| RFC3 | TTHERM_00213600 | 5.98 | <http://tfgd.ihb.ac.cn/search/detail/gene/TTHERM_00213600> |
| RFC4 | TTHERM_00780750 | 7.22 | <http://tfgd.ihb.ac.cn/search/detail/gene/TTHERM_00780750> |
| RFC5 | TTHERM_00161180 | 6.20 | <http://tfgd.ihb.ac.cn/search/detail/gene/TTHERM_00161180> |
| ATR | TTHERM_01008650 | NC | <http://tfgd.ihb.ac.cn/search/detail/gene/TTHERM_01008650> |
| RAD51 | TTHERM_00142330 | NC | <http://tfgd.ihb.ac.cn/search/detail/gene/TTHERM_00142330> |
| ASI2 | TTHERM_00191480 | NC | <http://tfgd.ihb.ac.cn/search/detail/gene/TTHERM_00191480> |
| CDT1 | TTHERM_00277530 | 7.79 | <http://tfgd.ihb.ac.cn/search/detail/gene/TTHERM_00277530> |

Z-score: In the Tetrahymena Gene Network (TGN) database (http://tfgd.ihb.ac.cn/tool/network), which can be used to identify related genes in the same biological processes or pathways, the Z-score was used in the context likelihood of relatedness (CLR) algorithm based on microarray expression data [24,37]. The larger of a Z-score between two genes indicated the more similar expression profiles.

NC: No correlation; Z-score is below the threshold 3.49.
